# Tidal Deformation and Dissipation Processes in Icy Worlds

**DOI:** 10.1007/s11214-025-01136-y

**Published:** 2025-01-16

**Authors:** G. Tobie, P. Auclair-Desrotour, M. Běhounková, M. Kervazo, O. Souček, K. Kalousová

**Affiliations:** 1 Laboratoire de Planétologie et Géosciences, UMR 6112, CNRS, Nantes Université, Université d’Angers, Le Mans Université, Nantes, France; 2 IMCCE, CNRS, Observatoire de Paris, PSL University, Sorbonne Université, Paris, France; 3 Faculty of Mathematics and Physics, Department of Geophysics, Charles University, V Holesšovičkách 2, Praha, Praha 8 180 00 Czech Republic; 4 Faculty of Mathematics and Physics, Mathematical Institute, Charles University, Sokolovská 83, Praha, Praha 8 186 75 Czech Republic

## Abstract

Tidal interactions play a key role in the dynamics and evolution of icy worlds. The intense tectonic activity of Europa and the eruption activity on Enceladus are clear examples of the manifestation of tidal deformation and associated dissipation. While tidal heating has long been recognized as a major driver in the activity of these icy worlds, the mechanism controlling how tidal forces deform the different internal layers and produce heat by tidal friction still remains poorly constrained. As tidal forcing varies with orbital characteristics (distance to the central planet, eccentricity, obliquity), the contribution of tidal heating to the internal heat budget can strongly change over geological timescales. In some circumstances, the tidally-produced heat can result in internal melting and surface activity taking various forms. Even in the absence of significant heat production, tidal deformation can be used to probe the interior structure, the tidal response of icy moons being strongly sensitive to their hydrosphere structure. In the present paper, we review the methods to compute tidal deformation and dissipation in the different layers composing icy worlds. After summarizing the main principle of tidal deformation and the different rheological models used to model visco-elastic tidal response, we describe the dissipation processes expected in rock-dominated cores, subsurface oceans and icy shells and highlight the potential effects of tidal heating in terms of thermal evolution and activity. We finally anticipate how data collected by future missions to Jupiter’s and Saturn’s moons could be used to constrain their tidal response and the consequences for past and present activities.

## Introduction

The discovery of huge volcanic eruptions on Io by Voyager 1 (Morabito et al. 1979), theoretically predicted by Peale et al. (1979) a few weeks prior to this historical flyby, confirmed that tidal dissipation can be a major driver for the evolution of the moons of the giant planets. The first observations of complex tectonic structures on Europa’s surface (Smith et al. 1979) also highlighted the role of tidal forces, potentially maintaining a subsurface ocean at shallow depths (Cassen et al. 1979). The encounter with the Saturn system also revealed an abnormal geological activity on the small moon Enceladus, for which the role of tidal heating was suspected (Squyres et al. 1983), even though there was a lot of doubt at the time about the feasibility of generating sufficient power to melt such a small satellite (Peale et al. 1980; Poirier et al. 1983). On Titan, no direct observation of its surface was possible, but its high orbital eccentricity, which cannot be forced by any other satellite, suggests that tidal friction was limited and has little influence on its evolution (Sagan and Dermott 1982; Sears 1995).

The first observations by Voyager 1 and 2 hinted at the possibility of subsurface oceans in icy moons and at the role of tidal processes in explaining any sign of surface activity. The confirmations were provided decades later by the more detailed exploration by Galileo (1995-2003) at Jupiter and Cassini-Huygens (2004-2017) at Saturn. Magnetic measurements performed by Galileo confirmed the presence of a subsurface salty ocean in Europa from magnetic induction (Khurana et al. 1998). On Europa, the presence of a subsurface ocean at a relatively shallow depth ($< 30$ km) beneath a tectonically active ice shell is attributed to tidal forces which may fluctuate on geological timescales due to mutual gravitational interactions with Io and Ganymede, through an orbital resonance, known as the Laplace resonance (e.g. Hussmann and Spohn 2004). The ice shell thickness and thermal state of Europa’s interior are thus expected to vary at a rhythm imposed by the Laplace resonance. The evolution of Europa is consequently intimately linked to that of Io and Ganymede.

Even more surprisingly, similar magnetic signatures were observed at Callisto and Ganymede (Kivelson et al. 2000; Zimmer et al. 2000), even though their interpretation as a signature of an electrically conductive subsurface ocean is more ambiguous due to the presence of a magnetic field generated in the iron core at Ganymede (Kivelson et al. 2002) and due to ionospheric effects altering the interpretation of magnetic induction of a subsurface ocean at Callisto (Hartkorn and Saur 2017). For Ganymede, the presence of a salty ocean has been confirmed from the observations of its oscillating auroral ovals by Hubble Space Telescope observations, whose limited oscillation amplitude implies an electrically conductive layer (Saur et al. 2015). On Callisto, we should wait for the exploration by the JUpiter ICy moon Explorer (Juice) to get confirmation of such an ocean (Van Hoolst et al. 2024). In addition to magnetic induction techniques, Juice will perform detailed measurements of tidal Love numbers $k_{2}$ and $h_{2}$ at Ganymede (De Marchi et al. 2022; Steinbrügge et al. 2015) and will determine with less precision the tidal Love number $k_{2}$ at Callisto (Cappuccio et al. 2022). Such measurements will provide key constraints on the hydrosphere structure and on the dissipation processes occurring in the different layers. The interpretation of these future data requires a detailed modeling of the tidal deformation and associated dissipation in these icy moons.

Thanks to Cassini gravity data, Titan is the first icy moon for which the tidal Love number has been estimated (Iess et al. 2012; Durante et al. 2019). The estimate of $0.616 \pm 0. 067$ published by Durante et al. (2019) can be interpreted as the signature of a global ocean at a depth between 50 and 100 km for an average ocean density between 1150 kg m^−3^ (weakly salty water) to 1270 kg m^−3^ ($\sim 15$ wt% salted ocean) (Mitri et al. 2014; Sotin et al. 2021). Idini and Nimmo (2024) proposed that the high value of the Love number reported by Durante et al. (2019) could be explained by a resonant, stably stratified ocean. More recently, by re-analysing the Cassini radio tracking data, Goossens et al. (2024) inferred a much smaller Love number, $0.375 \pm 0.06$, implying a less dense ocean below a thicker ice shell. It is still unclear which estimate is the most reliable. Moreover, the elevated orbital eccentricity of Titan (0.03) would imply limited dissipation in its interior during most of its history (Tobie et al. 2005a, 2006). However, Cassini data analysis suggests rapid orbital expansion of Titan implying much larger dissipation in Saturn than initially thought (Lainey et al. 2020). If such a rapid expansion is confirmed (which is still debated by Jacobson 2022), this would imply that Titan’s eccentricity would decay more slowly, thus allowing more dissipation inside Titan than what was previously predicted. It is also possible that Titan’s eccentricity was excited by interacting with another large moon, has disappeared since then, as proposed by Wisdom et al. (2022) to explain Saturn’s obliquity and the ring youth. A detailed analysis of dissipation processes inside Titan and the consequences for its orbital evolution is needed to better understand the recent evolution of the Saturnian system.

Probably the biggest surprise in space exploration in the last two decades were the discoveries of active plumes of water vapour and icy grains emitted from the south pole of Enceladus by the Cassini spacecraft in 2005 (e.g. Dougherty et al. 2006; Hansen et al. 2006; Porco et al. 2006; Spencer et al. 2006; Waite et al. 2006). Two decades after the first suspicion of eruption activity on Enceladus (e.g. Squyres et al. 1983), the observations made by Cassini demonstrated that tides were a key driver of the activity of Enceladus, leading to tidal heating dominating its heat budget in a way similar to Io. The concentration of activity at the south pole suggested the presence of an internal ocean decoupling the ice shell from the silicate interior (Nimmo et al. 2007), at least in the southern hemisphere (Tobie et al. 2008; Běhounková et al. 2012). The ocean was then confirmed to be global from the detection of a physical libration indicating an average ice shell thickness initially estimated between 20 and 25 km (Thomas et al. 2016; Van Hoolst et al. 2016) and recently re-evaluated between 27 and 33 km from a lower estimate of the libration amplitude (Park et al. 2024). The interpretation of long-wavelength topography and low-degree gravity field (Iess et al. 2014; Čadek et al. 2016; Beuthe 2016; Tajeddine et al. 2017; Čadek et al. 2019) further indicates that the ice shell is strongly thinned at the south pole, possibly thinner than 5 km. The intense activity observed on Enceladus is clearly related to tidal forces, but the processes at the origin of tidal heat production, and how the energy is concentrated to the south polar terrain and evolves through geological time is still strongly debated (e.g. Nimmo et al. 2007; Tyler 2009; Kite and Rubin 2016; Hemingway and Mittal 2019; Souček et al. 2019; Neveu and Rhoden 2019; Kang et al. 2022a; Nimmo et al. 2023).

**Fig. 1:**
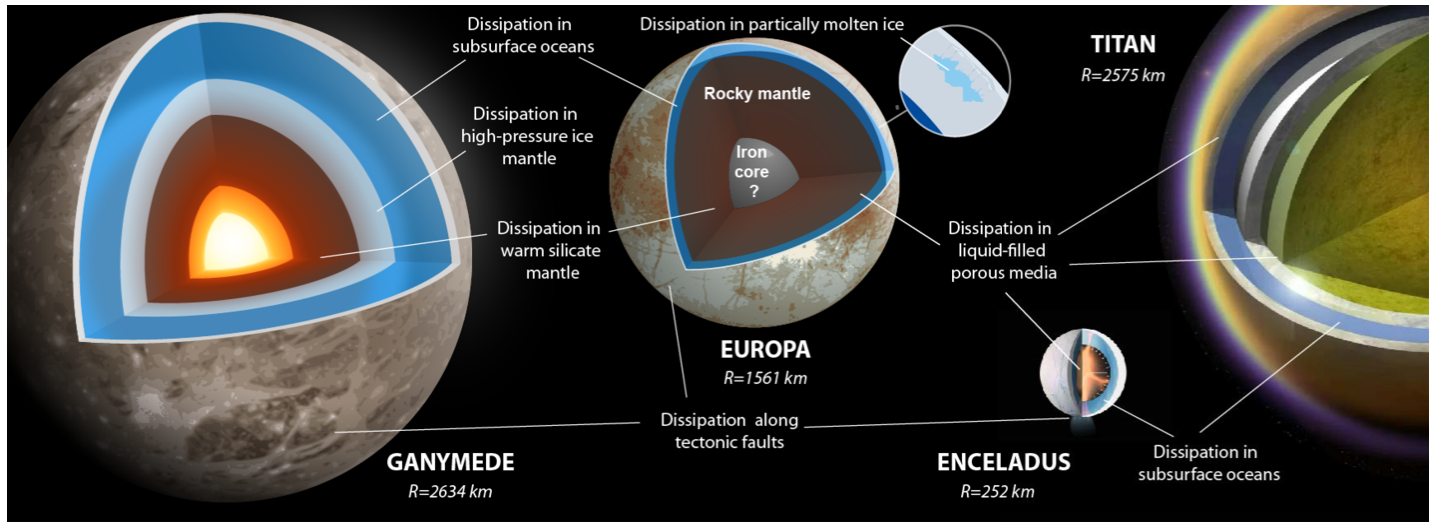
Schematic illustration of interior structure and possible dissipation processes in a selection of icy worlds

In this article, we review the main physical processes controlling the tidal deformation and dissipation in icy worlds (see Fig. 1) and the key controlling factors. We primarily focus on Enceladus and Europa, for which the most compelling evidence of tidally-induced processes has been observed. However, most of the processes discussed here are universal and may be relevant for many other icy worlds at present or during specific periods of their evolution. The content of this article is complementary to the article by Howett et al. (2025, this collection) dedicated to Enceladus heat budget and by Nimmo et al. (2023, this collection) dedicated to the tidally-controlled evolution of Enceladus. We also invite interested readers to consult previous reviews on similar topics, notably the synthesis of Hussmann et al. (2010) providing a broad overview on the energy sources and heat transport in icy worlds, and that of Bagheri et al. (2022a) providing complementary information on the tidal response of rocky and icy interiors. In Sect. 2, we present the general principle and computation technique to model tidal deformation and dissipation in planetary interiors. Section 3 describes the rheological properties of planetary materials composing icy world interiors, viscoelastic solids and partially molten rocks and ices. Section 4 is devoted to dissipation processes in rock-dominated layers and to the implications for hydrothermal and volcanic activities. Section 5 deals with tidal processes in subsurface oceans and coupling with the solid layers. Section 6 addresses the tidal response of icy shells, including convective ice shells, tidally-induced melting and porous flow in ices, and the tidal response of tectonic faults and water-filled cracks. Finally, Sect. 7 summarizes how future exploration missions may constrain tidal processes from geophysical measurements.

## Principle of Tidal Deformation and Dissipation

### Tidal Potential for a Satellite in 1:1 Spin-Orbit Resonance

The tidal forces exerted on a satellite in orbit around its central planet are the consequence of the difference in gravitational attraction created by the central planet throughout its satellite interior. As the gravitational force scales with $1/d^{2}$, $d$ being the distance between the planet and the satellite, it is consequently slightly weaker at the far side than at the near side of the satellite. For a satellite in a stable orbit around its planet, the average gravitational force exerted at the center of mass is compensated by the centrifugal force due to the orbital motions around the center of mass of the satellite-planet system. If we subtract the average gravitational force exerted on the center of mass of the moon, we obtain a residual force field, which tends to stretch the satellite in the central planet direction and to flatten the poles.

For a satellite in 1:1 spin-orbit resonance on a circular orbit, the gravitational force and hence tidal force would remain constant, as both satellite-planet direction and distance remain fixed. In reality, the satellite orbit is never perfectly circular, so that any small eccentricity of the orbit results in time variations of the tidal force due to modulation of planet-satellite distance and direction. The satellite can also have a non-zero obliquity relative to the orbital plane and any long-term variation in the rotation rate (long-period libration or secular drift), which also add to modulation of the tidal force.

The tidal force is conveniently described as the gradient of a scalar field $V_{T}$, called the tidal potential, which can be developed in several components as a function of radius, $r$, co-latitude, $\theta $, longitude, $\lambda $, and time, $t$ (e.g. Wahr et al. 2009; Jara-Orué and Vermeersen 2016):

1 $$ V_{T}(r,\theta , \lambda , t)=\frac{3GM_{P}R_{S}^{2}}{2a^{3}}\left ( \frac{r}{R_{S}}\right )^{2}\left [T_{*}+T_{0} +T_{1} + T_{2} + T_{3} \right ] $$

where

2 $$\begin{aligned} T_{*}=\frac{1}{6}\left (1-3 \cos ^{2}\theta \right ), \end{aligned}$$

3 $$\begin{aligned} T_{0}=\frac{1}{2}\sin ^{2}\theta \cos \left (2\lambda +2bt\right ), \end{aligned}$$

4 $$\begin{aligned} T_{1}=\frac{e}{2}\left (1-3\cos ^{2}\theta \right )\cos \left (nt \right ), \end{aligned}$$

5 $$\begin{aligned} T_{2}=\frac{e}{2}\sin ^{2}\theta \left [3\cos \left (2\lambda \right ) \cos \left (nt\right )+4\sin \left (2\lambda \right )\sin \left (nt \right )\right ], \end{aligned}$$

6 $$\begin{aligned} T_{3}=2 \sin (\theta )\cos (\theta )\sin (\epsilon )\cos (\lambda ) \sin (\omega +nt). \end{aligned}$$

with $a$, $n$, and $e$ the semimajor axis, the mean motion and eccentricity of the satellite orbit, $G$ the gravitational constant, $M_{P}$ the planet mass, $R_{S}$ the satellite radius, $b$ the angular rate of non-synchronous rotation, $\epsilon $ the obliquity of satellite’s spin axis, $\omega $ the argument of the pericenter measured with respect to the ascending node where the satellite’s orbital plane crosses its equatorial plane, and $t$ the time relative to the pericenter.

The term, $T_{*}$, describes the static flattening of the satellite, and $T_{0}$ describes the quasi-static stretching in the direction of the planet, which can slowly vary in the case of non-synchronous rotation. These two terms have no consequences for the heat budget of the satellite as they do not vary with time (or only very slowly). However, they control the shape of the satellite and the principal degree-two coefficients of the gravity field, which strongly depends on the density profile in the satellite interior and hence they are related to the moment of inertia of the satellite.

The terms $T_{1}$, $T_{2}$ and $T_{3}$, represent the diurnal tidal potential associated with orbital eccentricity and satellite obliquity, and are, therefore, responsible for the diurnal modulation of the satellite distortion. This modulation results in surface motions, internal mass redistribution and heat production by tidal friction.

### Methods for Computing the Tidal Response of Ice-Covered Ocean Worlds

Different modeling strategies have been proposed in the literature to compute the displacements and stresses associated with tidal perturbation, as well as the resulting tidal dissipation. The standard approach used in most published studies consists of solving the equations of motion and Poisson’s equation by assuming a spherically symmetric isotropic interior model. By neglecting any lateral variations in mechanical properties, the solutions can be separated into radial functions, classically denoted $y_{i}$, and spherical harmonics (e.g. Alterman et al. 1959; Takeuchi and Saito 1972), an approach commonly used to compute the spheroidal oscillation of self-gravitating interior. The set of equations to be solved can then be reorganized in a system of ordinary differential equations relying on six radial functions (noted $y_{i}$ following the notation of Takeuchi and Saito 1972), describing the radial and lateral displacement ($y_{1}$ and $y_{3}$), the radial and tangential stresses ($y_{2}$ and $y_{4}$), the potential ($y_{5}$), and a modified gravity function ($y_{6}$). This formulation initially developed for the elastic case can be extended to the visco-elastic problem by using the correspondence principle (Biot 1954), which allows for the computation of the equivalent elastic solution in the frequency domain with complex moduli. As long as an effective complex shear and bulk modulus (see Sect. 3) can be defined for each internal layer in the frequency domain, the equivalent solution can be computed. For more details on the equation derivation and their numerical resolution, see, for instance, appendix A in Roberts and Nimmo (2008) and Kervazo et al. (2021).

The advantage of this approach is that the equations can be solved numerically for any linear rheology, interior structure and complex rheology profiles. Once the radial functions are determined, the displacement, stress, and associated dissipation rate can be determined at any point inside the satellite interior. At the surface, the radial functions also give access to the so-called Love numbers, $k_{2}$, $h_{2}$, $l_{2}$, which are parameters quantifying the amplitude of potential perturbation, radial and lateral displacements, respectively, induced by tidal forces. These parameters can be directly related to the observed time variations of gravity field and surface displacements. The inconvenience of this efficient computation technique is that any lateral variation in layer thickness or mechanical properties cannot be taken into account self-consistently. Some approximations are, however, possible to mimic the effect of long-wavelength variations in ice shell thickness and viscosity structures.

Another approach consists of solving directly the governing equations for viscoelastic deformation in the time domain in a full 3D geometry. The problem can be solved either using a spectral approach or finite-element techniques. However, compressibility and self-gravity are usually neglected for the sake of simplicity. The solution is also usually limited to a single layer, and the interaction with other layers is considered by using the appropriate boundary conditions or by their parameterization. To take into account only lateral variations in viscosity, a spectral approach is probably the most appropriate. This has been proposed notably to study the potential role of a low viscosity zone above a regional sea at Enceladus’ south pole (Tobie et al. 2008; Běhounková et al. 2012). Such a spectral approach is particularly relevant for computing in a consistent way tidal dissipation from the 3D field of viscosity and hence allows for the full coupling with thermal convection codes (Běhounková et al. 2010, 2012, 2021) (see Sect. 6.1). To take into account other variations in mechanical properties, such as variations in ice shell thickness and local reduction of shear modulus associated with faults, a finite-element approach is required (Zhong et al. 2012; A et al. 2014; Souček et al. 2016, 2019; Berne et al. 2023). Such an approach is much more consistent, but highly demanding in computing resources. For viscoelasticity models, even though in theory any kind of rheological models could be considered, determining the viscoelastic memory term is computationally demanding, so an approximation consists in defining an effective viscosity using the Maxwell formalism but reproducing the dissipation rate expected for Andrade rheology (see Sect. 3). Alternatively, variations in shell thickness considering any viscoelastic rheology (though without any faults or any lateral variations in viscosity) can be treated with the thin shell approach of Beuthe (2018, 2019).

Once the different components of the stress field, $\sigma _{ij}$, and the strain field, $\epsilon_{ij}$, due to tidal perturbation are determined, the tidal dissipation rate, $h_{\mathrm{tide}}$, averaged over one tidal cycle, can be estimated at any point inside the satellite:

7 $$ h_{\mathrm{tide}} (r, \theta , \phi )= \frac{1}{T_{\mathrm{tide}}}\int _{t}^{t+T_{ \mathrm{tide}}}{\sigma _{ij}:\dot{\epsilon}_{ij}}{\mathrm{d}} t'. $$

In the frequency domain, this corresponds to:

8 $$ \tilde{h}_{\mathrm{tide}} (r, \theta , \phi )= -\frac{\omega}{2}\left [ \mathfrak{Im}(\tilde{\sigma}_{ij})\mathfrak{Re}(\tilde{\epsilon}_{ij})- \mathfrak{Re}(\tilde{\sigma}_{ij})\mathfrak{Im}(\tilde{\epsilon}_{ij}) \right ], $$

with $\omega =2\pi /T_{\mathrm{tide}}$, $\tilde{x}$ indicating Fourier transforms (Tobie et al. 2005b) and $\mathfrak{Re}()$ and $\mathfrak{Im}()$ corresponding to the real and imaginary parts of complex quantities, respectively. In this formulation, both shear and bulk dissipation can be included as long as both shear and bulk dissipative parts are defined (see Sect. 3.2 and Kervazo et al. 2021 for more details).

By integrating over longitude and latitude and taking into account both shear and bulk dissipations, and assuming a spherically symmetric structure, the radial distribution of the tidal dissipation rate can be determined by the radial sensitivity functions to shear and bulk moduli, $H_{\mu}$ and $H_{K}$, introduced by Tobie et al. (2005b) and determined from the radial functions $y_{i}$:

9 $$ \tilde{h}_{\mathrm{tide}}(r) = -\frac{21}{10} \frac{\omega ^{5}R_{s}^{4}e^{2}}{r^{2}}\left [H_{\mu }\mathfrak{Im}( \tilde{\mu})+H_{K} \mathfrak{Im}(\tilde{K})\right ], $$

with $\mu $ and $K$ the shear and bulk moduli, respectively. Note that the radial sensitivity functions of Tobie et al. (2005b) have been complemented by angular sensitivity functions in Beuthe (2013), which makes it easier to compute spatial patterns of dissipation. Once integrated over the whole interior, we can show that the global dissipated power, $P_{\mathrm{tide}}$, is then directly related to the imaginary part of the Love number, $\mathfrak{Im}(k_{2})$:

10 $$ P_{\mathrm{tide}} = -\frac{21}{2}\mathfrak{Im}(k_{2}) \frac{(\omega R_{s})^{5}}{G}e^{2}. $$

Note that the factors $\frac{21}{10}\,e^{2}$ and $\frac{21}{2}\,e^{2}$ in the above equations are based on the fact that 1:1 spin-orbit configuration is considered with a small eccentricity and neglecting the obliquity term. For any other orbital configuration, the appropriate factor should be re-derived for the corresponding tidal potential (see, for instance, Wisdom 2008; Efroimsky and Makarov 2014).

While the tidal response of solid bodies is particularly well-established and benefits from detailed constraints in the case of the Earth, the Moon or Mars, a particular challenge remains in the modeling of the tidal response of ice-covered ocean and its coupling with the ice shell. Models of tidal deformation based on the standard viscoelastic gravitational theory include the effect of the liquid layer. However, the dynamical response of such a layer is either considered in a simplified manner by ignoring the Coriolis effect, which breaks spherical symmetry (Takeuchi and Saito 1972; Kamata et al. 2015; Beuthe 2015b; Kervazo et al. 2021) or (most often) totally ignored by modeling the liquid layer as a static medium (Saito 1974; Sabadini et al. 1982; Spada 2008) or as a solid layer with a very low viscosity value (e.g. Nimmo et al. 2007), which ignored both inertial and Coriolis effects in the liquid layer.

In order to overcome these limitations, several authors adopted a more sophisticated formalism by self-consistently including the ocean-ice interactions into Laplace’s tidal equations (LTEs) (e.g. Beuthe 2016; Matsuyama et al. 2018) (see Sect. 5 for further details). The visco-elastic adjustment of the overlying shell and underlying solid interior can strongly affect the oceanic response by both attenuating and shifting the resonant peaks of the dynamical oceanic tide. These can cause orders of magnitude changes in the dissipated energy flux if the oceanic configuration is close to a resonance (e.g. Matsuyama 2014; Beuthe 2016). Recently, Aygün and Čadek (2023) showed that the dissipation rate obtained using the LTE approach corrected by the damping effect of the ice shell can be significantly different from that obtained by solving the three-dimensional Navier-Stokes equations taking into account viscoelastic coupling with the solid layers. This clearly indicates that the ocean response and the deformation of the solid part must be solved in a self-consistent way, in particular when the ocean configuration is close to a resonant state.

## Rheological Properties of Icy World Interiors

### Viscoelastic Rheological Models

**Fig. 2:**
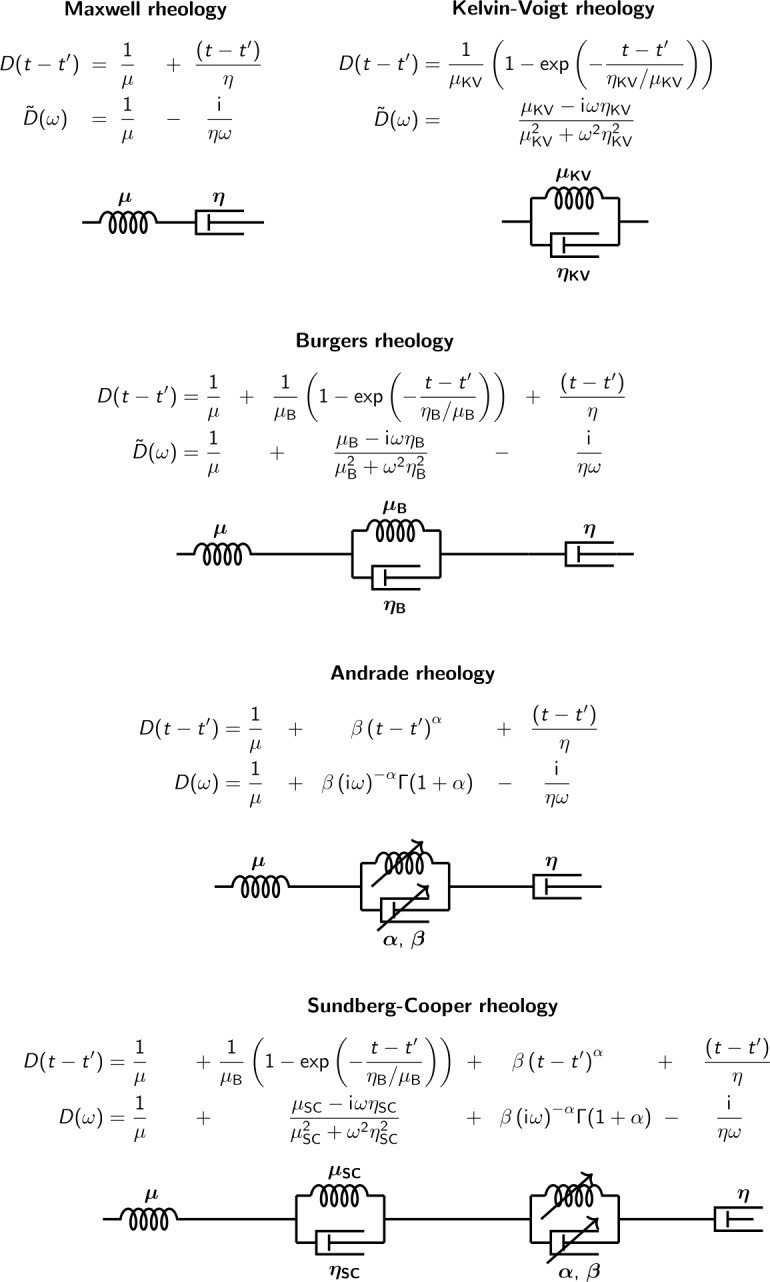
Schematic representation of rheological models for the shear component and corresponding dynamical compliance formulation in time and frequency domain. See the main text for the definition of the different notations used for the mathematical description of each rheological conceptual model. In the Andrade model, $\Gamma $ corresponds to the Gamma function defined as $\Gamma (z) = \int _{0}^{\infty} x^{z-1} e^{-x} dx$

At periods of tidal forcing (a few days for icy worlds), planetary interiors exhibit anelastic behaviour, which can be described as a combination of elastic and viscous responses. Deformation of the tidally perturbed body is delayed relative to the forcing, and energy is dissipated during the deformation cycle. Part of the deformation is purely elastic and is determined by the elastic shear and bulk moduli of the materials. The other part is anelastic, and is determined by the various attenuation mechanisms that can occur in planetary materials (ice, brines, organics, rock, partially molten rocks, iron, etc). Various rheological models have been proposed to describe the viscoelastic behaviour of planetary interiors. The simplest viscoelastic model, called the Maxwell model (Fig. 2), is classically represented by an elastic spring and a viscous piston (or damper) in series. From these simple elements, a large number of viscoelastic materials can be established by assembling them in series or in parallel (Fig. 2). While most studies devoted to modeling planetary tides were classically based on the Maxwell model, more complex models taking into account rheological behaviours observed in the laboratory on analogous materials are now being used to model the response of planetary interiors to tidal forcing (Henning et al. 2009; Castillo-Rogez et al. 2011; Behounková and Cadek 2014; Henning and Hurford 2014; Kuchta et al. 2015; Renaud and Henning 2018; Tobie et al. 2019; Bagheri et al. 2022a; Amorim and Gudkova 2024; Musseau et al. 2024; Bierson 2024).

Whatever the assumed rheological models, the relationship between the deviatoric/shear stress $\sigma '_{kl}$ and the deviatoric/shear strain $\epsilon '_{kl}$ during cyclic loading is conveniently described using a compliance function, $D(t)$, in the time domain (e.g. Jackson 2000):

11 $$ 2\epsilon '_{kl}(t)=\int _{-\infty}^{t}\dot{\sigma '}_{kl}(t')D(t-t')dt', $$

which corresponds to $\tilde{D}(\omega )$ in the frequency domain:

12 $$ 2\tilde{\epsilon '}_{kl}(\omega )=\tilde{\sigma '}_{kl}(\omega ) \tilde{D}(\omega ). $$

The dynamic compliance in the frequency domain is a complex variable, $\tilde{D}(\omega )=D_{1}(\omega )+\mathrm{i} D_{2}(\omega )$, from which the effective complex shear modulus can be directly calculated:

13 $$ \mathfrak{Re}({\mu _{\mathrm{eff}}}) = \frac{D_{1}}{D_{1}^{2} + D_{2}^{2}} \quad , \quad \mathfrak{Im}({\mu _{\mathrm{eff}}}) = - \frac{D_{2}}{D_{1}^{2} +D_{2}^{2}} $$

Here, we briefly review these different conceptual viscoelastic models and derive their dynamic compliance functions and effective complex moduli, required to compute the viscoelastic tidal deformation as described in Sect. 2, for each of these models.

#### Maxwell Model

**Fig. 3:**
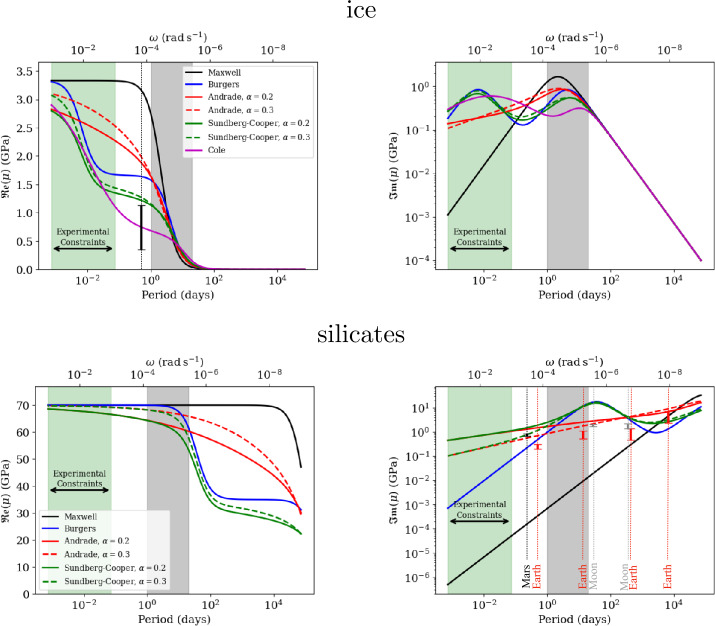
Comparison of predicted real (left) and imaginary (right) parts of the complex shear modulus as a function of period/frequency for the different rheological models presented in the text, for ices (top) and silicates (bottom). The green bands indicate the typical frequency/period at which experimental data exists, while the grey band indicates the typical range for tidal deformation. For ices, the elastic shear modulus is set to 3.3 GPa and the shear viscosity to $10^{14}$ Pa s, and for silicates to 70 GPa and $10^{20}$ Pa s, respectively. The $\alpha $ parameter in the Andrade model is set to 0.2 or 0.3 and $\beta $ to $1/\mu \tau _{M}^{\alpha}$. The parameters in the Cole model are set to: $\alpha _{g}=0.53$, $\tau _{d}=3300$ s, $\delta D=1.4\times 10^{-9}$ Pa^−1^. Burgers and Sundberg-Cooper parameters are set to: $\mu _{\mathrm{B}}=\mu _{\mathrm{SC}}=3.3$ GPa for ices, $\eta _{\mathrm{B}}=\eta _{\mathrm{SC}}=60$ GPa s and $\mu _{\mathrm{B}}=\mu _{\mathrm{SC}}=70$ GPa, $\eta _{\mathrm{B}}=\eta _{\mathrm{SC}}=7\cdot 10^{16}$ Pa s for silicates. As a comparison, the imaginary part of the shear modulus ($\sim 70/Q$ GPa) is provided using the Q factor estimates for Mars, the Earth and the Moon at different periods/angular frequencies (indicated by the vertical lines): Mars (black) at 5.5 hours, $Q=95\pm 10$ (Khan et al. 2018); the Moon (grey) at 28 days, $Q=38\pm 4$ (Williams and Boggs 2015), at 1 year, $Q=41\pm 9$ (Williams and Boggs 2015); the Earth (red) at 12.42 hours, $Q=295 \pm 65$ (Ray et al. 2001), at 13.66 days, $Q = 115 \pm 30$ (Ding et al. 2021), at 27.5 days, $Q = 85 \pm 25$ (Zou et al. 2024), at 433 days, $Q= 55 \pm 15$ (Zou et al. 2024), at 18.6 years, $Q= 40 \pm 20$ (Benjamin et al. 2006)

**Fig. 4:**
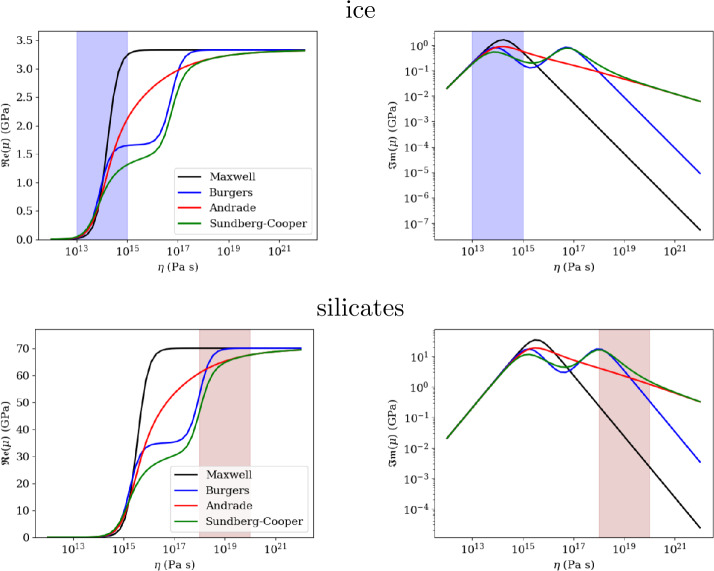
Comparison of predicted real (left) and imaginary (right) parts of the complex shear modulus as a function of viscosity for the different rheological models presented in the text, for ices (top) and silicates (bottom). The blue and pink bands indicate the typical range of viscosity near the melting point for ices and silicates, respectively. The tidal period is set to 3.55 days, corresponding to the case of Europa. All the other rheological parameters are similar to those used in Fig. 3

As mentioned above, the Maxwell model consists of an elastic spring and a viscous piston (or damper) in series. The spring, therefore, deforms instantaneously in response to applied stress while the piston slides gradually until the stress is relaxed. For a forcing period close to or larger than the Maxwell time, defined as the ratio of the shear viscosity $\eta $ to the shear modulus $\mu $, $\tau _{M} = \eta /\mu $, the Maxwell rheology is a good approximation. It fails, however, to reproduce correctly the dissipation function over a wide range of temperature (or viscosity), as it underestimates the dissipation rate with decreasing temperature (increasing viscosity) (e.g. Castillo-Rogez et al. 2011; Efroimsky 2012) (see Fig. 3 and 4).

#### Kelvin-Voigt Model

This model consists of a parallel arrangement of viscous damper and elastic spring elements; it is characterized by the retardation time $\tau _{\mathrm{KV}}=\eta _{\mathrm{KV}}/\mu _{\mathrm{KV}}$ describing the retardation of strain upon the application of constant stress. The Kelvin-Voigt model has the peculiarity of not having instantaneous behaviour and of producing a delayed elasticity effect, which makes it not really relevant to describe visco-elastic response at typical tidal frequencies. A Kelvin-Voigt element, however, describes the recoverable deformation of the medium and is considered to describe more complex viscoelastic models mentioned below, so it is important to introduce it here.

#### Burgers Model

This model corresponds to a Kelvin-Voigt element in series with the Maxwell element. The main advantage of this model is that it captures viscous creep and possible transient anelastic effects at forcing periods smaller than the Maxwell time, $\tau _{M}$. This transient effect is mimicked by adding a second damper in parallel with a second spring, associated with two additional parameters, $\mu _{B}$ and $\eta _{B}$.

#### Andrade Model

This rheological model is of particular interest for the tidally induced deformation of planetary bodies (e.g. Bierson 2024). This material can be seen as a Maxwell body to which an infinite number of springs and pistons are added in series. This extension, whose description is purely empirical, makes it possible to describe the body’s transient response in a more accurate way than the Burgers model, by introducing only two additional parameters, $\alpha $ and $\beta $, which can be assessed experimentally. The Andrade model accounts for a large range of experimental data on various metals and minerals (e.g. Jackson 2000). The parameter $\alpha $ typically varies between 0.1 and 0.5 (e.g. $\alpha =1/3$ in Jackson 2000) for olivine-rich rocks. It characterizes the duration of the transient phase of the first part (primary creep) of the material’s response and represents diffusion along single grains (Jackson et al. 2002). The parameter $\beta $ is related to the intensity of anelastic friction. Castillo-Rogez et al. (2011) have noted that, for various experimental data on olivine, $\beta $ is proportional to $1/(\mu \tau _{M}^{\alpha})$. Comparison with existing experimental data acquired at relatively high frequencies ($>10^{-3}$ Hz) indicates that $\beta /(\mu \tau _{M}^{\alpha })$ may typically vary between 0.1 and 10 (Walterová et al. 2023; Bierson 2024), however large uncertainties on this parameter still remain (e.g. Amorim and Gudkova 2024). The complex shear modulus displayed on Fig. 3 and 4 has been computed assuming $\alpha =0.2-0.3$ and $\beta /(\mu \tau _{M}^{\alpha })=1$ for the Andrade model.

#### Sundberg-Cooper Mode

Sundberg and Cooper (2010) discovered in their high-temperature olivine experiments that a Burgers-type attenuation peak connected to the elastically accommodated grain boundary sliding tended to appear in conjunction with an attenuation better characterized by the Andrade model. As neither the Burgers nor the Andrade formalism was able to account for this feature, they developed a composite rheological model combining the characteristics of both, known as the Sundberg-Cooper model.

#### Cole Model

For the particular case of water ice, a specific visco-elastic model has been proposed by Cole and Durell (1995) based on cyclic loading tests. These mechanical tests revealed that viscoelastic response is primarily controlled by dislocation motions in ice crystals and, therefore, is related to the density of mobile dislocations in crystals. As the other viscoelastic models presented above, the mathematical formulation of this model remains empirical, but it has the merit of relying on microphysical processes through the dislocation density. Dynamic compliance of the Cole model is written as:

14 $$ \tilde{D}^{C} (i \omega ) = \frac{1}{\mu} + \delta D\left [1- \frac{2}{\pi}\tan ^{-1}\left (\exp (\alpha _{g} s)\right )\right ] -i \delta D \frac{\alpha _{g}}{\exp (\alpha _{g} s)+\exp (-\alpha _{g} s)}- \frac{i}{\omega \eta}, $$

where $s= \ln (\tau _{d} \omega )$, $\tau _{d}$ is the relaxation time, $\alpha _{d}$ is a parameter describing the width of the relaxation peak, and $\delta D$ depends on the density of mobile dislocations, $\Delta $, a restoring stress, $\sigma _{r}$, acting on the dislocation motions, an orientation factor, $\Omega $, and the Burgers vector, $b$, such as $\delta D= \Delta \Omega b^{2}/\sigma _{r}$.

#### Comparison Between the Different Rheological Models and Existing Experimental and Geophysical Constraints

The Maxwell rheology has been widely used to calculate tidal heating in planetary interiors of outer Solar System satellites, either in their rocky mantle or core (e.g Hussmann and Spohn 2004; Tobie et al. 2005b; Moore and Hussmann 2009), or in the ice shell (e.g. Ojakangas and Stevenson 1989; Tobie et al. 2003). This model is suitable for describing the relaxation properties of materials when the forcing period is close to or above their Maxwell time. However, it does not allow one to quantify the attenuation of materials over a wide range of frequencies and temperatures. On Earth, this model has been successfully used to describe the relaxation process associated with postglacial rebound, which occurs on time scales of a few thousand years (e.g. Peltier 1974), but extrapolation of Maxwell’s model to tidal periods leads to dissipation values $Q^{-1}$ several orders of magnitude lower than the global value derived from observations (e.g. Egbert and Ray 2001; Sotin et al. 2009). The Maxwell model also fails to reproduce the anelastic response observed in experimental studies performed on rocks at the seismic frequency range, i.e. at frequency several orders of magnitude higher than the Maxwell frequency (e.g. Takei et al. 2014; Faul and Jackson 2015). As shown on Fig. 3, the imaginary part of the shear modulus, $\mathfrak{Im}(\mu )$, computed for a reference viscosity of $10^{20}$ Pa s, is 4 to 5 orders of magnitude lower than the values derived from global $Q^{-1}$ for the Earth (Ray et al. 2001) and Mars (Khan et al. 2018) when considering the Maxwell model. The Maxwell model leads to values comparable to observations only for the 18.6-yr lunar-nodal cycle. At shorter tidal periods, the Andrade and the Sundberg-Cooper models provide a dissipation rate more representative of the values inferred for Mars, the Earth and the Moon. This comparison is made assuming a single viscosity value of $10^{20}$ Pa s, while in reality more complex viscosity profiles should be considered for these three planetary bodies (Čížková et al. 2012; Karato 2010; Bagheri et al. 2019; Pou et al. 2022; Walterová et al. 2023). For each body, the viscosity profile and the rheological parameters should be adjusted to obtain a good fit. The main merit of the comparison displayed in Fig. 3 is to indicate which models between Andrade and Sundberg-Cooper provide a better representation of existing geophysical constraints. These models are also more suitable to reproduce the anelastic response of olivine and rock analogues at seismic frequencies (Jackson et al. 2002; Sundberg and Cooper 2010; McCarthy et al. 2011; Castillo-Rogez et al. 2011; McCarthy and Cooper 2016; Bierson 2024).

For ices, the most relevant geophysical constraints come from the tidal bending of Earth’s ice shelf margins in Antarctica and Greenland (e.g. Vaughan 1995; Reeh et al. 2003; Hulbe et al. 2016; Rosier et al. 2017; Wild et al. 2017). The tidal deflection records of floating glaciers suggested an effective shear modulus at tidal frequencies 3 to 10 times smaller than the elastic modulus of natural polycrystalline ices (Sinha 1989). This reduction in apparent modulus is likely the consequences of significant viscous attenuation (e.g. Reeh et al. 2003; Wild et al. 2017) and of reduced stiffness due to the presence of bottom crevasse and a firn layer at the surface (e.g. Rosier et al. 2017). Direct comparisons cannot therefore be made between laboratory measurements of ice elastic modulus and effective elastic modulus inferred from fitting models to ice-shelf flexure profiles. However, the inferred effective modulus provides key information on how natural ices behaves at tidal frequencies, for which no laboratory constraints exist yet. A few experimental studies investigated the viscoelastic response of natural or synthetic ice samples to cyclic loading (Tatibouet et al. 1986, 1987; Cole and Durell 1995; Cole 1998; McCarthy and Cooper 2016), but tests were performed at frequencies higher than $10^{-3}$ rad s^−1^. Mechanical tests revealed that the anelastic response of ice is related by the intracrystalline dislocation relaxation and to a lesser extent by grain boundary relaxation. Studies by Cole and Durell (1995) and McCarthy and Cooper (2016) indicated that the attenuation and shear modulus reduction is rather insensitive to grain size and appear mostly controlled by the density of mobile dislocations. Porosity, brine and crystal orientation also play a role by further enhancing attenuation and shear modulus reduction (Cole 1998). Mechanical tests by McCarthy and Cooper (2016) performed on fine-grained ice samples revealed a very dissipative behaviour, characterized by an abrupt shear modulus reduction and an strong increase of attenuation when frequency is reduced to $10^{-4}$ Hz. We can expect that shear modulus reduction and attenuation increase will be even more pronounced at tidal frequencies ($<10^{-5}$ Hz).

Among the different rheological models, only the experimentally-based Cole model predicts a significant shear modulus reduction with increasing periods (or decreasing frequencies) (Fig. 4, top left), which seems also consistent with the behaviour observed by McCarthy and Cooper (2016). The Maxwell and Andrade models totally fail to reproduce the expected shear modulus reduction, while Burgers and Sundberg-cooper models can reproduce it partly for periods lower than 0.1 days. As shown by Cole and Durell (1995) and McCarthy and Cooper (2016), the viscoelastic response of ice depends on a variety of microstructure properties, notably on the density of mobile dislocations. Following the Cole model, the shear modulus reduction and dissipation enhancement with decreasing frequencies is related at first order to the dislocation density in ice crystals. Interestingly, for the examples displayed on Fig. 4 (top left), we can notice that the shear modulus predicted by the Cole model is typically in the same range as the apparent shear modulus inferred by tidal bending of Earth’s floating glaciers. We should nevertheless keep in mind that the likely presence of crevasse should further reduce the apparent shear modulus (e.g. Nimmo 2004; Rosier et al. 2017) So more complex and realistic bending model should be used to infer the viscoelastic response of floating glaciers and to compare it to existing laboratory data on ice samples.

Extrapolation to icy moon conditions where tidal frequencies are $< 10^{-4}$ rad s^−1^ should be done with care. While the different rheological models converge to similar behaviour for the real part of the shear modulus at angular frequencies lower than $10^{-5}$ rad s^−1^ (Fig. 3, top left), they result in significant differences for the imaginary part, potentially up to one order of magnitude between the Cole and Maxwell models. Interestingly, the Cole model, which better reproduces the strong attenuation observed in laboratory data (Cole and Durell 1995; McCarthy and Cooper 2016), predicts the lowest dissipation at tidal frequencies.

In the absence of experimental data on the correct frequency range, it is difficult to conclude which model is the most appropriate to describe the viscoelastic response of ices and silicates in icy moon conditions. As shown by Bierson (2024), despite a wealth of experimental and seismic data, it is still difficult to conclude regarding the most appropriate rheological model and to determine the appropriate parameter values in order to correctly predict the tidal response of icy worlds. Due to its inability to reproduce experimental data and existing geophysical constraints, we can at least conclude that the Maxwell model is not appropriate. The Andrade model, which was originally developed to describe the deformation response of copper metal samples in the laboratory (Andrade 1910), shows promise for modelling transient creep in rock and ice (e.g. Faul and Jackson 2005; Sundberg and Cooper 2010; Faul and Jackson 2015; McCarthy and Cooper 2016; Bierson 2024). One of the features of Andrade’s rheology is the softening of the strong frequency dependence of Maxwell’s model, with a response characterized by a plateau, called an attenuation band in Andrade’s case contrasting sharply with Maxwell’s model, where the attenuation peak occurs at a mathematically exact frequency, with a sharp drop on either side. Such a reaction is different from a purely viscous response whose details are lost after the load is removed (irreversible), retaining some aspect of material“memory” (which can be reversible or irreversible, Efroimsky 2012). This memory depends not only on the static properties of the material (as is the case with the Kelvin-Voigt model), but also on how the microphysical properties of the material under consideration have evolved over time. On the other hand, one of the drawbacks of Andrade’s model is that its two additional parameters are not directly associated with classical material property values such as viscosity or shear modulus, as is the case with Maxwell rheology, and which have to be assessed from laboratory data. By compiling a wide range of experimental data on rocks and analogous materials, Bierson (2024) showed that the Andrade parameters, $\alpha $ and $\beta $ are poorly constrained. In particular, the $\beta $ parameter may vary by several orders of magnitude, which may imply much more dissipative interiors that initially anticipated and affect the link between dissipation rate and viscosity (Amorim and Gudkova 2024).

Sundberg and Cooper (2010) proposed that the serial combination of an Andrade mechanism and Burgers rheology, now considered as the Sundberg-Cooper model, resulted in a better fit for laboratory data on olivine at high frequency ($> 10^{-3}$ Hz). The experiments of Sundberg and Cooper (2010) are of particular value to the planetary community, as they were conducted both with samples of mantle-like materials and at mantle-relevant temperatures. However, it is still unclear if the extrapolation of such models to lower frequency regimes predicts the correct dissipative function. The applicability of such a model to icy materials is also an open issue. Several experimental studies on rocks (e.g. Sundberg and Cooper 2010; Faul and Jackson 2015) and ices (e.g. Cole and Durell 1995; McCarthy and Cooper 2016) have revealed the complexity of the viscoelastic response of planetary materials by showing how sensitive it is to temperature, grain size, dislocation density, impurity concentration etc. There is still a lot of work to do to identify the correct rheological law at low-frequency regime.

Whatever the assumed rheological model, a peak of dissipation in ices is expected for viscosity values near the melting point ($10^{13}-10^{15}$ Pa s, Fig. 4). This has major consequences for the thermal state of the ice shell as further discussed in Sect. 6.1. Interestingly, a second dissipation peak predicted by Burgers and Sundberg-Cooper models for viscosity values of the order of $10^{17}$ Pa s which may provide an additional source of tidal heating closer to the surface, in the conductive lid and along active faults. Nevertheless, we should keep in mind that this secondary peak is a direct consequence of the mathematical formulation of the Burgers model and the Sundberg-Cooper model (which includes a Burgers element), it is not guaranteed that this formulation predicts the correct viscoelastic behaviour of ices at low frequency and low temperature. For silicates, these secondary peaks may also significantly increase the dissipation rate for viscosity values near the melting point ($\sim 10^{18}$ Pa s). This secondary peak, far from the Maxwell relaxation peak, corresponds to the peak observed at higher frequencies for silicate minerals at high temperatures and should be associated with similar relaxation mechanisms. As shown by Renaud and Henning (2018), this secondary peak, if really occurring, could significantly impact the tidal evolution of the rock interior of planetary bodies.

In summary, several empirical viscoelastic models have been proposed. Some of them reproduce, in a satisfactory manner, existing laboratory data obtained on cyclic loading tests, unfortunately at frequencies significantly higher than the typical tidal frequencies. Extrapolation of empirical functions to planetary conditions significantly different from those at which parameters have been derived in the laboratory should be considered with care when modeling the tidal response.

### Bulk Dissipation

All the viscoelastic models presented above considered only shear deformation and stress as a source of mechanical dissipation. Global-scale seismic attenuation models of Earth indicate that bulk dissipation is small compared to shear dissipation (e.g. Widmer et al. 1991; Durek and Ekström 1996; Romanowicz and Mitchell 2015). As a consequence, bulk contribution has been classically ignored in studies dedicated to tidal deformation of planetary bodies. However, it might be non-negligible in some circumstances, in particular for partially molten interiors, such as Io (Beuthe 2013; Kervazo et al. 2021). Bulk dissipation can be considered by adding a term reflecting the bulk viscosity $\zeta $ associated with volumetric deformation, in addition to the standard shear viscosity associated with shear deformation. The constitutive equation relating stress $\sigma _{kl}$ and strain $\epsilon _{kl}$, including both the shear and bulk terms, of a Maxwell rheology is then written:

15 $$ \dot{\sigma}_{kl} + \frac{\mu}{\eta} \Bigg(\sigma _{kl}-\frac{1}{3} \sigma _{ii} \delta _{kl}\Bigg) + \frac{K}{\zeta} \frac{1}{3} \sigma _{ii} \delta _{kl}= 2 \mu \dot{\epsilon}_{kl} + \Bigg(K- \frac{2}{3} \mu \Bigg) \dot{\epsilon}_{ii} \delta _{kl}, $$

where $\delta _{kl}$ is the Kronecker symbol. The Fourier transform of Equation (15) leads to a relationship similar to Hooke’s law for the purely elastic case:

16 $$ \tilde{\sigma}_{kl} = \tilde{\lambda}(\omega ) \tilde{\epsilon}_{ii} \delta _{kl} + 2 \tilde{\mu}(\omega ) \tilde{\epsilon}_{kl} $$

with

17 $$ \tilde{\lambda}(\omega )= \tilde{K}(\omega ) - \frac{2}{3} \tilde{\mu}(\omega ) , $$

18 $$ \tilde{\mu}(\omega ) = \frac{\mu \omega ^{2} \eta ^{2}}{\mu ^{2}+\omega ^{2}\eta ^{2}} + i \frac{\mu ^{2}\omega \eta}{\mu ^{2}+\omega ^{2}\eta ^{2}} , $$

19 $$ \tilde{K}(\omega )= \frac{K \omega ^{2} \zeta ^{2}}{K^{2}+\omega ^{2} \zeta ^{2}} + i \frac{K^{2} \omega \zeta}{K^{2}+\omega ^{2} \zeta ^{2}}, $$

where $\omega $ is the frequency, $\eta $ the viscosity, $\mu $ the shear modulus, $K$ the bulk modulus and $\lambda $ the Lamé parameter. The tilde indicates the Fourier transform. These choices of $\tilde{\lambda}$, $\tilde{\mu}$ and $\tilde{K}$ assume that viscoelastic relaxation occurs for both shear deviatoric $\sigma '_{ij}$ and bulk $\sigma _{nn}$ stresses. In this case, unlike the pure shear case, the bulk modulus $K$ is also affected and differs from the elastic case.

**Fig. 5:**
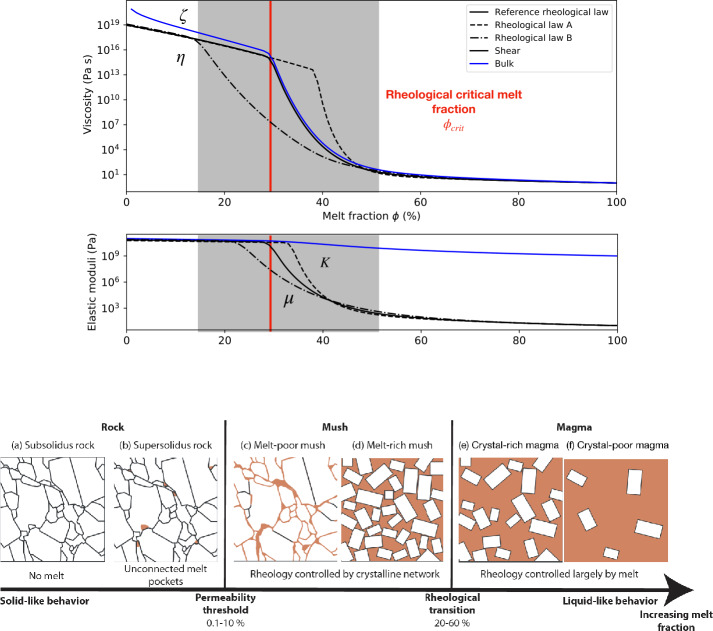
*Top*. Effect of melt fraction $\phi $ on the viscoelastic parameters of a rocky mantle: shear viscosity $\eta $ and modulus $\mu $ (in black); bulk viscosity $\zeta $ and modulus $K$ (in blue). The three models correspond to three different rheological transitions where the transition between solid-state and liquid-state behaviors occurs. The red vertical line corresponds to the rheological critical melt fraction RCMF for the reference model (here $\phi _{\mathrm{crit}}=30$%). The “solid” shear viscosity (for $\phi = 0$), used as a reference value is that of silicate rocks close to their melting point, typically of the order of $10^{19}$ Pa s (Karato and Wu 1993). The liquid value (for $\phi = 1$) of the shear viscosity is of the order of 1 Pas, as observed for basaltic melts (e.g. Shaw 1969). For the mantle conditions typical of Io and Europa, the solid bulk modulus $K$ is generally between 150 and 250 GPa. The solid shear modulus $\mu $ of a silicate mantle is typically 60 GPa. Concerning the values corresponding to the liquid case, ultrasonic velocity measurements indicate that for basaltic liquids, $K$ is an order of magnitude lower than the value expected for mantle minerals, between 1 and 30 GPa (e.g. Murase and McBirney 1973). Figure adapted from (Kervazo et al. 2021). *Bottom*. Schematic illustration of possible physical configurations of melting in a partially molten silicate mantle. The ranges for permeability and rheological transition thresholds are uncertain and depend in particular on rock composition. Figure adapted from Sparks et al. (2019)

The dissipated power per unit volume averaged over one orbital is then determined by both $\mathfrak{Im}(\tilde{\mu})$ and $\mathfrak{Im}(\tilde{K})$ as (e.g. Beuthe 2013):

20 $$ \tilde{h}_{tide}(r,\theta ,\phi )=\omega \mathfrak{Im}(\tilde{\mu}) \left (\tilde{\epsilon}_{kl}\tilde{\epsilon}^{*}_{kl}-\frac{1}{3}| \tilde{\epsilon}|^{2}\right )+\frac{\omega}{2}\mathfrak{Im}(\tilde{K})| \tilde{\epsilon}|^{2}, $$

with ∗ indicating a complex conjugate. Most of the time, $\mathfrak{Im}(\tilde{\mu})\gg \mathfrak{Im}(\tilde{K})$, so that bulk dissipation can be neglected. However, for partially molten materials, as shown hereafter, $\mathfrak{Im}(\tilde{K})$ can become comparable to $\mathfrak{Im}(\tilde{\mu})$ as $\mu $ rapidly decreases with increasing melt fraction to an asymptotic zero value, while the bulk modulus $K$ remains comparable to the solid phase even for a purely liquid phase (Fig. 5).

### Role of Melts on the Rheology of Rocks and Ices

Melting is a first-order phase change that has drastic consequences on the rheological properties of the materials that build planetary interiors, as well as on their dynamics, on different spatial and time scales. Under certain temperature and pressure conditions, a heterogeneous material, such as a planetary mantle, can become multiphase as a result of partial melting. The presence of interstitial melts in rocks is known to affect both their elastic (e.g. Budiansky and O’Connell 1976; Mavko 1980; Takei 1998; Faul et al. 2004) and viscous (e.g. Hirth and Kohlstedt 1995a,b; Kohlstedt and Zimmerman 1996; Scott and Kohlstedt 2006) properties. At a given point in the partial melting process, the material changes from a solid matrix with fluid pores to a fluid solution with isolated floating crystal grains. When the grains lose contact with each other, the material loses most of its shear strength and is controlled by the viscous properties of the fluid (Fig. 5). The transition from solid-state creep rheology to melt-dominated creep rheology, i.e. from solid to fluid behaviour of the material, led to the concept of a rheological critical melt fraction (RCMF) associated with a sharp transition from the solid behaviour to the liquid behaviour (e.g. Renner et al. 2000). In practice, most theoretical and experimental studies devoted to the effect of partial melts on the rheology of rocks focus on viscosity as this parameter plays a prominent role in the dynamics. Following the pioneering work of Arzi (1978), suggesting a threshold value of about 25–30% for RCMF, a compilation of theoretical and experimental studies applied to a solid undergoing partial melting, gives a wide range of values for this critical fraction, from 26 to 62% (e.g. Vigneresse et al. 1996; Renner et al. 2000; Scott and Kohlstedt 2006; Costa et al. 2009). Another important aspect of partially molten rocks is the possible contribution of bulk dissipation. Once above the rheological critical melt fraction, small changes in melt fraction can have a large impact on the dissipation rate, and feedback between melt-induced tidal heating and melt production may be amplified by the bulk viscoelastic response (Kervazo et al. 2021).

#### Constraints on Viscous Parameters

The relationship between the (shear or bulk) viscosity of partially molten rock and the rate of partial melting is of critical importance in characterizing the rheological behaviour of silicate mantles. In partially molten materials undergoing diffusion creep, the presence of melt leads to a reduction in the material’s effective shear viscosity compared to the situation without melt. One reason for this is that diffusion in the melt phase is generally much faster than in the solid phase (e.g. Takei and Holtzman 2009). Since the rate of diffusion creep depends on the rate at which material is transported at the grain scale, the presence of fast melting paths accelerates the overall rate of creep for a given stress. The extent of this effect depends essentially on the geometry of the grain-scale melting. The simplest model of grain-scale melting geometry is that of textural equilibrium, a state that minimizes surface energy. Furthermore, it should be noted that the complete description of a linear isotropic medium requires the use of two viscosities, implying a bulk viscosity, $\zeta $, in addition to the shear viscosity, $\eta $, (e.g. McKenzie 1984; Scott and Stevenson 1986; Ricard et al. 2001). Theoretical considerations indicate that bulk viscosity $\zeta $ decreases with increasing melt fraction and may become comparable to shear viscosity $\eta $ for melt fraction exceeding 10-20% (Schmeling et al. 2012). However, although viscosity has been the subject of more work than any other rheological property of natural silicate melts, there are no measurements of the bulk viscosity of natural or analogous systems applicable to the crust or mantle. Consequently, in large-scale geodynamic models, bulk viscosity is generally given by a law based on theoretical considerations (e.g. Scott and Stevenson 1986; Schmeling et al. 2012). For water ices, a few experimental studies have studied the effect of interstitial water on the viscosity of water ice at the melting point (De La Chapelle et al. 1999; Adams et al. 2021). Even a small amount of interstitial water can reduce significantly the effective viscosity of ice when approaching the melting point (Goldsby and Kohlstedt 2001; Adams et al. 2021). Based on the experimental work of (De La Chapelle et al. 1999), 5% of water content should result in a decrease of viscosity by one order of magnitude.

#### Constraints on Elastic Parameters

Unlike viscosity, the elastic properties of partially molten rocks have been studied only for small values of the melt fraction (typically a few percent). On Earth, this is indeed motivated by the existence of regions in the upper mantle where low seismic velocities and high attenuation are observed, which is interpreted as the presence of a small amount of partial melts. Theoretical models were developed to describe the effect of melt fraction on the shear and bulk modulus (Mavko 1980; Schmeling 1985). These models quantify the dependence of seismic wave speeds and attenuation upon melt fraction and fluid-filled inclusions of specified shape (e.g., ellipsoids, grain boundary films, or grain-edge tubes). The viscoelastic behaviour of partially molten rock has also been investigated in the laboratory through forced torsional oscillation (Berckhemer et al. 1982; Bagdassarov and Dingwell 1993; Faul et al. 2004), on rock or glass; as well as ultrasonic studies of shear and compression waves (e.g. Dingwell and Webb 1989).

For water ice containing interstitial brines, a few studies have quantified the effect of water content on the elastic and attenuation properties of ice (e.g. Spetzler and Anderson 1968; Schwarz and Weeks 1977; McCarthy et al. 2019). As an example, according to Spetzler and Anderson (1968), for ice containing 2% NaCl, the shear modulus can drop by a factor of 2 even at a temperature as low as −20 °C which corresponds to brine fraction of about 6-7%.

### Rheology of Unconsolidated Porous Materials

The rocky core of small icy moons, like Enceladus and other small moons, as well as the outer part of the rocky core of larger icy bodies likely exhibits a high degree of porosity due to the limited pressure (e.g. Vance et al. 2007; Roberts 2015). The low density of Enceladus’s rock core (2450-2600 kg m^−3^) as inferred from Cassini gravity data (Iess et al. 2014; Čadek et al. 2016; Beuthe et al. 2016), indicates a porosity of 20-30% (Choblet et al. 2017). Such a highly porous medium can be viewed as a highly fragmented/weakly cohesive material, where tidal deformation is likely associated with inter-granular friction during grain rearrangements or/and frictional sliding along microcracks (e.g. Seed et al. 1986; Ishibashi and Zhang 1993; Rollins et al. 1998; Wulff et al. 1999). Both mechanisms depend on the microstructure characteristics (grain distribution, microcrack density), which are unknown in the context of the icy moons’ interiors. However, the mechanical behaviour of fragmented, unconsolidated media, has been intensively studied in laboratories in the context of geotechnical applications. Even though existing mechanical tests are usually performed at frequencies significantly higher ($> 10^{-2}$ Hz) than tidal frequencies, these measurements can be used to provide some mechanical constraints in planetary contexts.

The mechanical response of highly fragmented/granular media is classically parameterized using the effective shear modulus, $\mu _{\mathrm{eff}}$, and the dissipation function, $Q_{\mu}^{-1}$ (equal to twice the damping ratio, a quantity classically derived in laboratory mechanical tests), which control the response amplitude to cyclic tidal forcing and the fraction of mechanical energy that is converted into heat, respectively (e.g. Seed et al. 1986; Ishibashi and Zhang 1993; Rollins et al. 1998; Wulff et al. 1999; Brennan et al. 2005). An effective viscosity could also be defined assuming a Maxwell rheology as it is classically done when computing tidal deformation in viscoelastic bodies (e.g. Tobie et al. 2005b; Roberts 2015). However, it is more meaningful to directly represent the dissipation rate as a function of effective shear modulus and dissipation function (or damping ratio) as these are the quantities inferred directly from mechanical tests in the laboratory.

The real and imaginary parts of the complex shear modulus, $\tilde{\mu}$, used to compute the tidal deformation, are determined from the effective shear modulus, $\mu _{\mathrm{eff}} =|\mu _{c}|$, and the local dissipation function, $Q_{\mu}^{-1}$, as follows:

21 $$ \mathfrak{Re}(\tilde{\mu})=|\tilde{\mu}|\sqrt{1-\left (Q_{\mu}^{-1} \right )^{2}}, \mathfrak{Im}(\tilde{\mu})=|\tilde{\mu}|Q_{\mu}^{-1}. $$

The forcing frequency is expected to affect both shear modulus and dissipation function, especially in weakly cohesive unconsolidated materials, which may be representative of the water-filled porous rocky core of Enceladus as argued by Choblet et al. (2017). As explained by Shibuya et al. (1995), the damping ratio (dissipation function) in cohesive soils is expected to: (i) increase at frequencies below 0.1 Hz, due to creep effects of soil skeleton; (ii) remain more or less constant between 0.1 and 10 Hz; and (iii) increase at frequencies greater than 10 Hz because of the pore fluid viscosity. Cyclic loading tests performed on unconsolidated granular materials, i.e. mixture of sands and/or gravels (e.g. Seed et al. 1986; Rollins et al. 1998; Zhou et al. 2017) at low confining pressure, indicate that the effective shear modulus strongly decreases for cyclic strain exceeding 0.01-0.1%, for forcing frequency ranging between typically 0.01 and 1 Hz. These mechanical tests also demonstrate that the damping ratio increases with decreasing shear modulus, and can reach values of about 0.3-0.4, corresponding to a dissipation function of 0.6-0.8. While the occurrence of this reduction of shear modulus (and increase of dissipation) is observed in all samples in laboratory tests (e.g. Rollins et al. 1998; Brennan et al. 2005), the exact values of cyclic strain at which the change of mechanical behavior occurs may depend on the characteristics of the sample (grain size distribution, composition, porosity etc.), as well as on the frequency and strain history. The confining pressure also influences the dissipation rate, but this effect remains moderate (e.g. Zhou et al. 2017) at least for confining pressures up to 5 MPa, corresponding to the pressure at the surface of Enceladus’ core. Unfortunately, no data exists at higher pressure to confirm this tendency. Even though the conditions inside Enceladus’ core are different from those considered in laboratory tests (higher frequency, lower pressure), Choblet et al. (2017) assumed that a similar trend should occur when the core materials are subjected to cyclic strain. This assumption should be confirmed by future mechanical tests in the appropriate range.

In the absence of direct constraints, we can estimate the core mechanical properties from the total power required to explain the global ocean underneath a relatively thin ice shell. By computing the tidal dissipation in Enceladus’ core, we show that the generation of 10-30 GW by tidal friction in Enceladus’ unconsolidated core requires an effective shear modulus ranging between $10^{7}$ and $10^{8}$ Pa and a dissipation function between 0.2 and 0.8. For such a weak and dissipative core, the cyclic strain in the core would range between 0.005 and 0.015%. According to the data of Seed et al. (1986) and Rollins et al. (1998), the dissipation function typically ranges between 0.05 and 0.2 for gravelly soils subjected to a cyclic strain of 0.01% in amplitude at a frequency ranging between 0.1 and 1 Hz. No mechanical tests exist at very low frequencies ($< 10^{-5}$ Hz) relevant for tidal deformation, however, we anticipate the dissipation function to be higher and the reduction of shear modulus to be more pronounced than at 0.1-1 Hz. At very low frequency ($< 0.01$ Hz), dissipation is indeed expected to increase due to creep effects in the solid matrix (e.g. Shibuya et al. 1995). The likely presence of clays or organics in the porous core could favor this creep effect and increase dissipation. Although performed on materials of a different nature, such an increase of dissipation and shear modulus reduction are observed in mechanical tests performed at very low frequencies ($10^{-4}-10^{-3}$ Hz) on polycrystalline aggregates (olivine, e.g. Faul and Jackson 2005 or ice, e.g. Cole and Durell 1995). Moreover, the cyclicity of tidal forcing may also result in strain accumulation and gradual build-up of pore pressure, favoring the weakness of the core material and maintaining it in a highly deformable state.

The tidally-induced motions of fluids in the porous unconsolidated rock matrix may also add additional dissipation as proposed by Liao et al. (2020), Rovira-Navarro et al. (2022) and Kamata (2023). Such dissipative processes, which can be estimated in the framework of the poroviscoelastic theory developed by Biot (1956), are controlled by the permeability and the viscosity of the fluids and are strongly frequency dependent. Efficient dissipation is expected to occur when the fluid phase oscillates in the solid matrix at frequencies approaching a characteristic frequency, $f_{c}$ defined by Biot (1956) as:

22 $$ f_{c} = \frac{\eta _{\mathrm{liq}}\phi ^{2}}{2\pi \rho _{\mathrm{liq}} k}, $$

where $\eta _{\mathrm{liq}}$ is the viscosity of the liquid phase, $\phi $ the porosity, $\rho _{\mathrm{liq}}$ the density of the liquid phase and $k$ Darcy’s coefficient of permeability. The dissipation due to periodic fluid motions in the pores starts to get non-negligible for frequency larger than 0.01 $f_{c}$. For typical values of liquid viscosity ($10^{-3}-10^{-2}$ Pa s), the porosity of $20-30$%, permeability of $10^{-14}-10^{-12}$ m^2^ (Choblet et al. 2017), and water density of 1000 kg m^−3^, dissipation would become non-negligible for frequencies larger than 300 Hz, so eight orders of magnitude larger than typical tidal frequencies ($\sim 10^{-5}$ s^−1^). As shown by Rovira-Navarro et al. (2022), significant dissipation could occur at tidal frequency only if the porous core was highly permeable with permeability of $10^{-5}$ m^2^ allowing high-amplitude Darcian flow and associated dissipation comparable to what is estimated on Enceladus ($\ge 10$ GW). But under such conditions, heat transfer out of the core would be extremely efficient at preventing any temperature increase in the core and any production of hot hydrothermal vents. For reasonable ranges of rock permeability, tidally-induced fluid motions are expected to be limited to the outer part of the porous core (depth $< 25$ km, Kamata 2023), leading to limited dissipation. The inner part of the core (depth $>25$ km) should remain undrained, with almost no fluid motions in the pore and hence no fluid dissipation.

## Tidally-Driven Hydrothermal and Volcanic Activities

### Tidal Heating and Heat Transport in Water-Saturated Porous Core

As proposed by Roberts (2015) and further explored by Choblet et al. (2017), the silicate core of mid-sized moons, such as Enceladus, could be highly fragmented, consisting either of rock fragments embedded in an ice matrix as long as the internal temperature remains below the melting point of water ice, or of fragmented porous rock core filled with liquid water. Shortly after accretion, the interior of such small moons is expected to be frozen (Monteux et al. 2014) and to remain in this state until the interior reaches the melting point due to progressive warming produced by both radiogenic and tidal heating. As shown by Neveu and Rhoden (2019), depending on the size of the moon, its rock fraction and possible orbital resonance encounters, it may take from a few hundred million years to a few billion years before the ice melts and leading the formation of a water-filled porous rock core.

As shown by Roberts (2015) in the case of Enceladus, the maximum dissipation rate in a core composed of an ice-rock mixture is estimated to be about $10^{-7}$ W m^−3^, while, for a water-filled porous core, the dissipation rate may exceed $10^{-6}$ W m^−3^ (Choblet et al. 2017). Tidal dissipation in an ice-rock interior is estimated to be at maximum of the order of 2 GW, which is not sufficient to explain the observed activity on Enceladus (Roberts 2015). In contrast, a water-filled fragmented porous core may generate more than 10 GW, for mechanical properties comparable to typical unconsolidated granular materials (Choblet et al. 2017, see also Sect. 3.4).

For an initially frozen interior, ice melting may be triggered when the moons pass through orbital resonances (e.g. Meyer and Wisdom 2008; Nakajima et al. 2019). During such periods of increased eccentricity, progressive warming until the melting point is expected to result in an enhancement of tidal heating, as viscosity and shear modulus get reduced, thus potentially leading to runaway melting until the whole icy interior melts and forms discrete fragmented porous core surrounded by a thick water ocean (Noyelles et al. 2019).

**Fig. 6:**
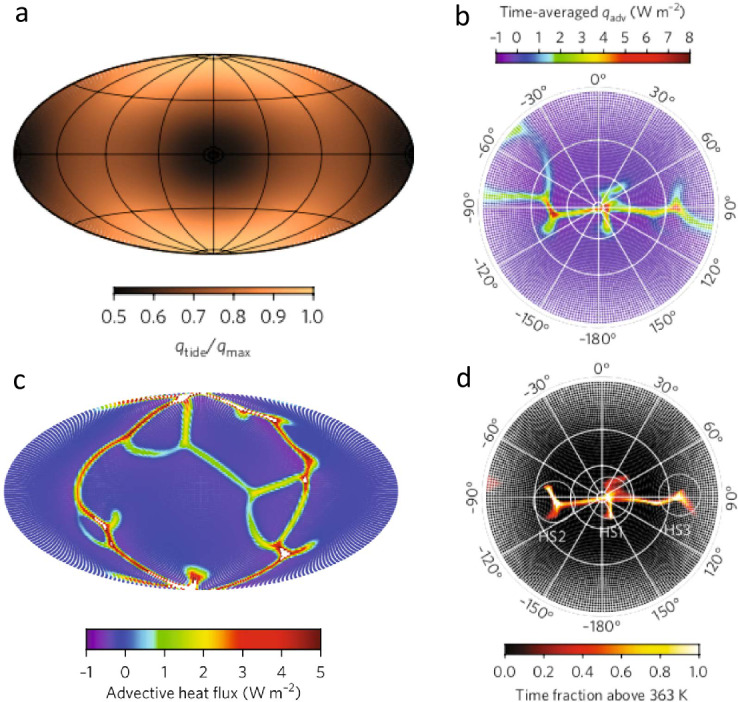
a) Spatial distribution of tidal heat flux in the porous rock core of Enceladus; b) polar view distribution and c) global distribution of the advective heat flux through the surface of the porous core due to tidally-driven hot water flow, for a total power of 30 GW and a core permeability of $10^{-13}$ m^−2^; d) Time fraction during which a given location at the rocky core/ocean interface remains at a temperature above 363 K (stereographic projection) considering a total simulation duration of 10 Myr (adapted from Choblet et al. 2017)

In the case of Enceladus, Choblet et al. (2017) showed that tidal heating in a weakly consolidated water-filled porous core should result in a warming of interstitial water powering efficient upward porous flow of hot water. The degree-two shape of the tidal potential results in a modulation of tidal heating as a function of latitude and longitude, with maximal tidal heating at the poles and along two specific meridians at 90° and −90° relative to the sub-/anti-Saturnian meridian (Fig. 6a). This moderate change in tidal heating results in a strong concentration of hot narrow upwellings in polar regions and along these meridians (Fig. 6c). These upwellings can sustain powerful (1–5 GW) hotspots at the seafloor, thus providing conditions for hydrothermal activities in the south polar region with water as hot as 363 K for core permeability between $10^{-14}$ and $10^{-13}$ m^−2^ (Fig. 6b, d). Such a concentration of heat release can thus explain the ice shell thinning observed at the south pole (Čadek et al. 2016; Beuthe et al. 2016; Čadek et al. 2019) and the associated jet activity (Porco et al. 2006, 2014). As long as the core remains weakly consolidated and permeable, such endogenic activity may be sustained, and could be operating for tens of millions to billions of years.

Similar tidally-heated water flow processes may also operate in other mid-sized moons, at least in the past (e.g. Castillo-Rogez et al. 2018). For instance, on Mimas, there is evidence for an ocean at present (Tajeddine et al. 2014; Lainey et al. 2024), which, based on the observation at Mimas’s orbital pericenter drift, would be at relatively shallow depths (20-30 km). If Mimas has a weakly consolidated porous core with mechanical properties similar to Enceladus, strong tidal dissipation is expected, potentially of the order of 10-20 GW (Lainey et al. 2024). The absence of any sign of endogenic activity at its surface suggests that such a highly dissipative state is geologically recent (Rhoden 2023; Rhoden et al. 2024; Lainey et al. 2024). Lainey et al. (2024) showed that the ocean, if present, must have started forming between 5 and 15 Myr ago when the eccentricity was about 2.5 times higher than at present, which may have been the result of resonance crossings with other Saturn’s moons (e.g. Ćuk and El Moutamid 2022; Ćuk et al. 2024, this collection). This result implies that Mimas might be an emerging ocean world, for which the timescale of ocean formation is directly controlled by the timescale of eccentricity damping (Lainey et al. 2024; Rhoden et al. 2024). The Mimas example highlights how crucial the understanding of orbital evolution is to better evaluate the past evolution of the icy moons. We refer interested readers to the articles by Nimmo et al. (2023) and Ćuk et al. (2024) in this collection for details on the long-term orbital evolution of the Saturnian system and the coupling with tidal dissipation.

Other moons, such as Saturn’s moon Dione or Uranus’s moon Ariel and Miranda, may also pass by a very dissipative state involving large heat production in a porous core and associated hydrothermal activity, as suggested from evidence of enhanced heat flux in the past based on crater relaxation (White et al. 2017; Hussmann et al. 2019; Beddingfield et al. 2022a,b). For larger bodies, Neptune’s moon Triton (e.g. Gaeman et al. 2012), Pluto (e.g. Bagheri et al. 2022b) and Eris (e.g. Nimmo and Brown 2023), tidal dissipation and porous flow may also have played a role, but most likely during their early stage just after differentiation, before core consolidation and compaction which should be much faster due to higher pressure compared to much smaller moons like Enceladus and Mimas. Future work is needed to evaluate how such a dissipative state of their porous rocky core, even if brief, may have influenced the physico-chemical evolution of these bodies and the habitability of their internal oceans.

### Coupling Between Tidal Heating and Convection: Implications for Magmatic Activities

Unlike smaller satellites where a significant fraction (if not all) of the rocky core can remain porous and unconsolidated during most of the satellite evolution, larger satellites such as Europa, Ganymede, Titan and Triton are expected to have a compact interior due to higher pressure and high temperature in the deep interior. Due to their large size, more energy accumulates during accretion and radiogenic heat is less efficiently removed from the deep interior than in smaller satellites where water transport in porous media is extremely efficient to limit internal temperature increase. For large moons like Europa and Triton, some porosity may be preserved in the silicate crust (Vance et al. 2016), but the depth at which water may circulate in non-compacted porous media probably does not extend 25-50 km. For even larger moons like Ganymede and Titan, the pressure at the base of the hydrosphere is probably too high to maintain any significant porosity. Below the porous upper crust, heat transfer will be dominated by heat conduction and advection similar to heat transfer in the Earth’s mantle.

The thermal evolution of the rock mantle can be described by the equations characterizing heat transfer by convection and/or conduction or two-phase flow if melt is present. Tidal dissipation combined with radiogenic heating enters the energy conservation equation and represents the primary heat source for long-term evolution. While the contribution of radiogenic heating inevitably decays over time, tidal dissipation remains a dynamic energy source influenced by orbital parameters. In the case of multi-body systems, where mean-motion resonances can come into play, tidal dissipation may represent a highly variable and significant source of energy (e.g. Hussmann and Spohn 2004).

The precise ratio of the two contributions remains uncertain. For Io, the observed heat budget (65 - 125 TW; e.g., Lainey et al. 2009) significantly exceeds estimates for radiogenic heating (Hussmann et al. 2010). In contrast, for Europa’s silicate mantle, radiogenic heating prevails, especially during the early phases of evolution. Only in the later stages of evolution and during periods of increased eccentricity is tidal dissipation speculated to become dominant (Běhounková et al. 2021). Tidal dissipation is expected to be even lower for Ganymedes’s mantle due to its distance from Jupiter (Bland et al. 2009; Steinbrügge et al. 2023). However, even in this case, the tidal dissipation in the rock mantle could have played a key role in the past when Ganymede entered the Laplace resonance. For eccentricity two times larger in the past, tidal heating in Ganymede could have been comparable to present-day tidal heating in Europa’s mantle, which can reach a level similar to radiogenic power (Steinbrügge et al. 2023).

Tidal energy is influenced not only by orbital parameters but also by the total mechanical responses of the moons. As explained in Sect. 2, the amplitude of tidal deformation is linked to the internal structure, while energy dissipation is sensitive to thermal conditions. In cases of extreme tidal dissipation, thermal runaways can occur, potentially initiating large-scale silicate melting causing an extreme volcanic activity as observed on Io (e.g., Lopes et al. 2004; Mura et al. 2020). This sensitivity of material properties to temperature and the presence of melt (see Sect. 3.3), gives rise to feedback between thermal evolution and tidal dissipation.

The interplay between thermal evolution and tidal heating becomes evident when we consider the sensitivity of the local tidal dissipation rate to temperature and viscosity (Fig. 4). Generally, the dissipation rate at tidal frequencies increases with increasing temperature and, consequently, with decreasing viscosity. This is a key characteristic that leads to positive feedback between the temperature and the magnitude of tidal dissipation.

Once the melting point is reached, the subsequent evolution depends on how efficiently the melt is extracted. In the extreme scenario, where the melt is instantaneously extracted to the mantle surface, the thermal runaway stops and any produced heat is balanced by melt extraction (Běhounková et al. 2021). Moore (2001) have proposed for Io the heat pipe mechanism, an equilibrium scenario in which heat produced by tidal dissipation is brought to the surface by the ascent of magma (see also Bierson and Nimmo 2016 and Steinke et al. 2020). In general, the melt ascent is described by the two-phase flow approach (see 1D two-phase approach for Io, Spencer et al. 2020, 2021), and can rapidly vary in time resulting in a disequilibrium between heat production and heat release.

Depending on the efficiency of melt extraction, significant melt can stay embedded in the solid matrix, affecting the mechanical properties of the molten region. The material begins to melt and gradually builds a system of interconnected channels. Once the local melt fraction reaches the disaggregation point (typically for 20-40% of melt, see Fig. 5), the rigidity is strongly affected and sharply decreases. Consequently, the tidal dissipation drops and the response to tidal loading changes (Moore 2001; Kervazo et al. 2021). Combined with strongly decreased viscosity and more effective heat transport, a new equilibrium near the disaggregation point could be theoretically reached.

The retention of melt, however, introduces an additional level of complexity (see also Sect. 3.3). A model of melt percolation within a solid-melt mixture (Miyazaki and Stevenson 2022) applied to Io suggests that the partial-melt layer ($\phi >0.2$) rapidly separates into two phases, creating a subsurface magma ocean. In cases where significant melt is retained, resulting in the formation of a magmatic ocean or the presence of a substantial amount of melt approaching critical porosity, mechanical separation of layers may occur. This separation can induce alterations in global deformations, which may be detectable. Moreover, analogous effects to those considered in oceans can also come into play (see Sect. 5). For instance, in the case of the emergence of a thin magma ocean, dissipation within such a fluid layer can pass through resonance phases connected to an increase in tidal dissipation, potentially accelerating the melting process (Tyler et al. 2015). However, it is important to note that the feedback with orbital evolution remains a subject of ongoing research and is not yet fully understood.

The distribution of melt and the location of its production remains uncertain, even in the case of Io where, in the absence of a hydrosphere, significant volcanic activity gives direct insight into the melting of its mantle (e.g. Hamilton et al. 2013; de Kleer et al. 2019; Davies et al. 2024). Magnetic induction also indicated a subsurface molten layer containing a melt fraction of 20% or more (Khurana et al. 2011). The spatial distribution of the melt production is linked to the temperature profile and the mechanisms governing melt transport. A significant factor influencing melt distribution is the presence or absence of convective heat transfer. In situations where convection is absent, melt originates in the deepest regions of the mantle, just above a possible core where the temperature is the highest. Conversely, in the presence of convection, the subadiabatic temperature gradient is reached (the temperature is slightly higher under the stagnant lid than above the lower boundary layer) consistent with convection with significant internal heating characteristics. Moreover, the melt production beneath the lithosphere due to the decompressive melting is expected (e.g., Steinke et al. 2020; Běhounková et al. 2021).

**Fig. 7:**
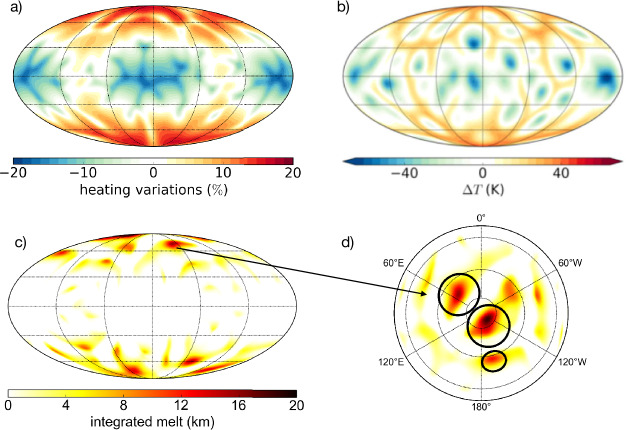
Example of 3D simulations of tidally-heated thermal convection in Europa’s mantle showing enhancement of tidal heating at high latitudes (a), resulting in a concentration of hot thermal plumes (b) and melt generation (c, d) towards the poles (adapted from Běhounková et al. 2021)

Due to tidally-induced changes in polar flattening and stretching in the planet direction, the maximum local heating rate is expected in the polar areas (provided the liquid layer lies beneath the deforming solid layer), and the minima are located in the equatorial area at sub- and anti-planet points in the case of a spin-orbit resonance configuration. Again, the presence of a fully liquid magma layer (Tyler et al. 2015) or a subsurface partially molten layer (e.g. Segatz et al. 1988; Tackley et al. 2001; Beuthe 2013; Bierson and Nimmo 2016; Steinke et al. 2020) maybe associated with bulk dissipation (Kervazo et al. 2021) can change this pattern substantially. At shorter wavelengths, dissipation is modulated by the temperature pattern originating in convection, and it is typically higher in the hot upwellings and lower in the cold downwellings. Conversely, tidal dissipation can break the relatively uniform distribution of convective planforms, can help focus the hot upwellings in the areas with the highest dissipation, and create sheet-like hot structures near the bottom boundary. For instance, in the case of Europa’s silicate mantle, it has been shown that enhancement of tidal heating toward the poles tends to maintain hot upwellings at high latitudes, leading to higher melt production towards the poles (Fig. 7). For eccentricity twice higher than the present-day value (conditions potentially reached during the last 100 million years (Hussmann and Spohn 2004)), the volume of generated melts during magmatic episodes could be comparable to those involved in the Large Igneous Provinces observed on Earth (Běhounková et al. 2021). Similar tidally-induced magmatic events may occur on the seafloor of other icy moons, especially during periods of enhanced eccentricity, for instance on Titan, Ganymede or Triton.

The evidence of past or ongoing magmatic activity at Europa’s seafloor relies on indirect observations as it is concealed under the icy shell. Gravity field measurements offer a potential tool to assess topographic gravity anomalies originating from volcanic activity (Pauer et al. 2010; Dombard and Sessa 2019; Koh et al. 2022; Mazarico et al. 2023). The anticipated primary contributors to the gravity field include the topography of the silicate/ocean and ice/ocean interfaces, along with surface topography (Koh et al. 2022). In the case of Europa, with its relatively thin hydrosphere, seafloor topography dominates the gravity signal up to a degree of $l=22$ (wavelength $>200$ km), making long-wavelength signatures of potential volcanic activity at the seafloor accessible to Europa Clipper (Koh et al. 2022). However, the seafloor gravity signal may be diminished if an additional putative layer containing materials like gypsum (Melwani Daswani et al. 2021) or carbonaceous matter (Reynard and Sotin 2023) is present. For Ganymede, the detection of possible (though unlikely) volcanic or tectonic activity is more challenging due to the attenuation of the signal with distance, the rock/ice interface being about 800 km below the surface (De Marchi et al. 2021).

## Tidal Friction in Subsurface Oceans

### The Equilibrium and Dynamical Tides

As indicated in Sect. 2, both solid and liquid layers in planetary interiors are subject to the gravitational tidal forcing generated by neighbouring bodies. However, the tidal response of the latter strongly differs from that of the former. The tidal dynamics of solid bodies is associated with long viscoelastic relaxation timescales (of the order of days for temperate ice layers and of the order of ${\sim }10^{3}{-}10^{4}$ yr for rock mantles typically; see e.g. Karato and Spetzler 1990; Tobie et al. 2003; Castillo-Rogez et al. 2011; Sabadini et al. 2016; Bolmont et al. 2020). As a consequence, one can neglect inertial effects, which refer to the dynamical effects induced by the motion of particles (advection, Coriolis effects). The solid tidal response thus takes the form of a slightly delayed quasi-static viscoelastic adjustment, as described by the viscoelastic-gravitational theory (Takeuchi and Saito 1972; Tobie et al. 2005b, 2019) (see Sect. 2.2). This behaviour is equivalent to the large scale hydrostatic adjustment of fluid layers characterising the low-frequency limit $\sigma \rightarrow 0$, with $\sigma $ being the forcing tidal frequency, which corresponds to the equilibrium tide (e.g. Ogilvie 2014). In the general case, however, the tidal response of fluid layers is frequency-dependent due to the excitation of waves, as detailed further. These waves form the so-called dynamical tide (Zahn 1975). As they are resonant for specific frequencies (or equivalent thicknesses of the fluid layer), they strongly affect the amplitude of the tidal fields and the resulting tidal dissipation.

In the equilibrium tide limit, a subsurface ocean can be treated as a non-elastic solid in the framework of the viscoelastic-gravitational theory (Beuthe 2015a,b). Under this approximation, the dynamical tide is filtered owing to the absence of inertial effects. As discussed by Kamata et al. (2015), although this static ocean formulation appears to be very convenient to compute the tidal response of multi-layered bodies as a whole, it delivers significant inaccuracies if the ocean thickness is not much larger than the resonant thicknesses associated with the excited tidal modes. One should then take into account the full dynamical oceanic response and the associated wave families. The only exception is the regime of ‘creeping’ flow where dissipative mechanisms are so strong that all inertial effects vanish. We note that Tyler et al. (2015) provide an analytical solution to the oceanic tidal equations in this creeping flow limit to model the tidal response of Io’s suspected global magma ocean (Khurana et al. 2011).

The simplest framework that allows the dynamical tide to be examined is the thin-shell approximation, which consists in ignoring the vertical structure of the fluid layer and the dependence of the tidal fields upon the radial coordinate, thus leading to the shallow water equations (e.g. Vallis 2017). In this approach, the ocean is considered as a thin spherical shell of incompressible fluid with two-dimensional tidal fields reflecting the integrals over the layer, which is valid as long as the ocean depth $H$ is much smaller than its distance to the centre of the body. The radial velocity is therefore assumed to be much smaller than the horizontal velocity, which makes it possible to ignore all the physical terms involving the vertical component of the velocity field in the momentum and continuity equations. We note that horizontal flows produce a vertical Coriolis acceleration that is not necessarily negligible with respect to pressure forces. However, this Coriolis acceleration induces substantial mathematical complications by coupling the vertical and horizontal structures of tidal flows together. It is therefore usually ignored by means of the traditional approximation, the limitations of which are thoroughly discussed by Gerkema and Shrira (2005) and Gerkema et al. (2008). This formalism has been widely used to study the tidal response of subsurface oceans in icy moons (e.g. Tyler 2011, 2014; Chen et al. 2014; Matsuyama 2014; Beuthe 2016; Hay and Matsuyama 2017; Matsuyama et al. 2018; Rovira-Navarro et al. 2023) and ocean planets (e.g. Webb 1980; Auclair-Desrotour et al. 2018; Motoyama et al. 2020; Tyler 2021; Farhat et al. 2022).

In the linear theory of tides, the tidal fields are considered as small perturbations with respect to the background fields of the body at rest. In particular, the surface elevation oscillations generated by the tidal forcing are assumed to be much smaller than the ocean depth, while their wavelengths are comparable to the radius of the body. Therefore, the non-linear advection of tidal flows is neglected. This yields the classical Laplace’s tidal equations (LTE) (e.g. Longuet-Higgins 1968; Lamb 1993). The LTEs are the mass conservation and horizontal momentum equations given by (e.g. Tyler 2011; Matsuyama 2014)

23 $$\begin{aligned} \partial _{t} \xi + \nabla \cdot \left ( H \boldsymbol{u} \right ) & = 0, \end{aligned}$$

24 $$\begin{aligned} \partial _{t} \boldsymbol{u} + 2 \boldsymbol{\Omega} \times \boldsymbol{u} & = - g \nabla \xi + \nabla U - \boldsymbol{F}_{\mathrm{drag}}, \end{aligned}$$

where $\xi $ designates the tidal variation of the ocean’s thickness, $\boldsymbol{u}$ the associated horizontal velocity field, $g$ the gravity, $U$ the forcing tidal gravitational potential, $\boldsymbol{\Omega}$ the spin vector, and $\boldsymbol{F}_{\mathrm{drag}}$ the drag force exerted on the layer per unit mass. The notations $\partial _{t}$, $\nabla $, and $\nabla \cdot $, refer to the partial derivative with respect to time, to the horizontal gradient operator, and to the horizontal divergence operator, respectively. In the case of spherical or hemispherical ocean basins of uniform depth, the LTEs admit closed form solutions where the tidal fields are expanded in terms of the oceanic normal modes, also called free oscillations (Longuet-Higgins 1966, 1968; Longuet-Higgins and Pond 1970). These modes are associated with specific eigenfunctions and the corresponding eigenfrequencies.

In the simplest configuration where the ocean is global and inviscid (no drag forces), the eigenfunctions are called the Hough functions (Hough 1898) and commonly denoted by $\Theta $ (e.g. Lindzen and Chapman 1969; Lee and Saio 1997; Wang et al. 2016). The Hough functions sharing the same order^1^$m$ are obtained by solving, for $\left ( \Theta , \Lambda \right )$, the eigenvalues-eigenvectors problem formulated as (e.g. Lee and Saio 1997)

25 $$ \left \{ \frac{d}{d \mu} \left [ \frac{1 - \mu ^{2}}{1- \nu ^{2} \mu ^{2}} \frac{d}{d \mu} \right ] - \frac{1}{1 - \nu ^{2} \mu ^{2}} \left ( \frac{m^{2}}{1-\mu ^{2}} + m \nu \frac{1 + \nu ^{2} \mu ^{2}}{1-\nu ^{2} \mu ^{2}} \right ) \right \} \Theta = - \Lambda \Theta , $$

assuming regularity boundary conditions at the poles. In the above equation, $\mu = \cos \theta $, where $\theta $ refers to the colatitude, and the real parameter $\Lambda $ is the eigenvalue associated with $\Theta $. Besides, $\nu = 2 \Omega / \sigma $ designates the spin parameter, which depends on the spin angular velocity, $\Omega $, and the forcing frequency, $\sigma $, and quantifies the impact of rotation on the forced modes (e.g. Lee and Saio 1997). In the static limit ($\nu = 0$), the Hough functions converge towards the associated Legendre functions of the spherical harmonics, while rotational effects become stronger as $\nu $ increases in absolute value.

Several authors use this simplified theory to obtain analytical tidal solutions for the torque exerted on global or hemispherical oceans with free-surface conditions (e.g. Auclair-Desrotour et al. 2018, 2019; Motoyama et al. 2020; Farhat et al. 2022). However, more realistic configurations including non-linearities and more complex geometries require to switch to numerical modelling. In those configurations, the equations governing the tidal dynamics are efficiently solved using spectral methods, which consist in expanding the tidal fields in series of spherical harmonics as a first step, and in calculating the spherical harmonic coefficients by inverting a set of algebraic equations as a second step (Longuet-Higgins 1968; Tyler 2011, 2014; Chen et al. 2014; Matsuyama 2014; Matsuyama et al. 2018; Beuthe 2016). Such methods are, for instance, implemented in the Tidal Response of Planetary Fluids (TROPF) software package (Tyler 2019). Other approaches seem, nevertheless, more suitable in the case of complex boundary conditions (spatially-varying ocean depth, topography), such as the finite element method employed in the Ocean Dissipation in Icy Satellites (ODIS) model (Hay and Matsuyama 2017, 2019) or in most of state-of-the-art Earth oceanic tidal models (e.g. Egbert et al. 1994, 2004; Green et al. 2017; Schindelegger et al. 2018; Daher et al. 2021).

### Several Families of Resonantly Excited Tidal Waves

In the tidal flows described by the shallow water approach, the fluid wave modes that carry the energy are divided into rotational-gravity and Rossby waves. The rotational-gravity and Rossby waves are sometimes referred to as the ‘class I’ and ‘class II’ oscillations, respectively (e.g. Longuet-Higgins 1968; Tyler 2021). The rotational-gravity waves refer to long-wavelength surface gravity waves modified by rotation. They have as a restoring force the gravity force exerted on the mass surplus resulting from an increase of the ocean surface elevation, and their typical phase speed is $c = \left ( g H \right )^{1/2}$ (Vallis 2017). Each mode is associated with an eigenfrequency $\sigma _{\mathrm{w}}$, and an equivalent depth $h = H \left ( \sigma / \sigma _{\mathrm{w}} \right )^{2}$, the latter referring to the ocean depth for which the mode would be resonant at the considered tidal forcing frequency (Taylor 1936; Hendershott 1981). The rotational-gravity waves exist for all values of the spin parameter $\nu $ introduced in Eq. (25) (Lee and Saio 1997). As $\nu \rightarrow 0$ (static limit), their spatial structure converges towards the spherical harmonics, which corresponds to pure gravity modes. Conversely, if $\left | \nu \right | \gg 1 $ (low-frequency tidal forcing), the rotational-gravity waves are confined within an equatorial band. Their spatial structure is thus strongly altered by rotation in this configuration. The role played by rotational-gravity waves in the tidal energy dissipation has been examined both in the context of the subsurface oceans harboured by icy satellites (e.g. Tyler 2011, 2014; Chen et al. 2014; Matsuyama 2014; Matsuyama et al. 2018; Beuthe 2016; Rovira-Navarro et al. 2023) and that of the Earth’s surface ocean (e.g. Webb 1980, 1982; Auclair-Desrotour et al. 2018; Tyler 2021; Farhat et al. 2022). As discussed further, these waves may be responsible for resonant amplifications of tidal heating in icy satellites, each mode being associated with a resonance occurring for $\sigma = \sigma _{\mathrm{w}}$ or, equivalently, for $h = H$.

Rossby waves, also called ‘Rossby-Haurwitz’ waves or ‘planetary waves’ (e.g. Longuet-Higgins 1968; Tyler 2021), are induced by rotation, their restoring force being the variation in Coriolis acceleration across the latitude. These waves can be excited by the tidal gravitational forcing if $\left | \nu \right | >1$ (Lee and Saio 1997). While they are confined near the poles if the effect of rotation is weak, they tend to span large regions of the globe as $\nu $ increases. As Tyler (2008) indicates it in the case of Europa, large-amplitude Rossby waves can be resonantly excited by tidal forces due to obliquity – although these forces are subdominant – because their eigenfrequencies are close to obliquity forcing frequencies. This statement equally applies to other moons with suspected subsurface oceans sharing the same tidal regime, such as Titan and Enceladus (Hay and Matsuyama 2017; Matsuyama et al. 2018; Rovira-Navarro et al. 2020, 2023).

The rotational-gravity and Rossby waves described in the framework of the thin-shell approximation form the barotropic flow of the tidal response, namely the component for which the density variation is a monotonic function of the pressure variation (Vallis 2017). These surface waves are thus associated with uniform motions of fluid particles over the water column. The vertical structure of the fluid layer ignored in the thin-shell approximation is characterised by the stability of the stratification with respect to convection (Gerkema and Zimmerman 2008). In stably stratified regions, fluid particles moving across the vertical direction tend to be driven back to their initial positions by the Archimedean force, whereas they are free to go away in neutrally stratified (or unstratified) regions. The Archimedean force thus acts on particles as a spring whose stiffness is quantified by the squared Brunt-Väisälä (or buoyancy) frequency, $N^{2} = g^{2} \left ( \partial \rho _{0} / \partial p_{0} - c_{\mathrm{s}}^{-2} \right )$, with $g$, $\rho _{0}$, $p_{0}$, and $c_{\mathrm{s}}$ designating the local gravity, density, pressure, and sound speed, respectively (e.g. Gerkema and Zimmerman 2008). In Earth’s ocean, $N$ varies from ${\sim} 10^{-4}~{\mathrm{s^{-1}}}$ in the deepest layers to ${\sim} 10^{-2}~{\mathrm{s^{-1}}}$ in the 200 m-region beneath the surface (e.g. Gerkema et al. 2008).

The upward-travelling waves that have the Archimedean force as a restoring force are called internal gravity waves. They develop in stably stratified water columns and form the baroclinic flow of the tidal response, where the tidal density and pressure fields varies non-monotonically across the vertical direction (Vallis 2017). Similarly to surface waves, internal waves are associated with equivalent depths and eigenfrequencies characterising their resonances. It is noteworthy that this baroclinic response completely vanishes if the ocean is unstratified ($N = 0$), which yields a pure barotropic response. Nevertheless, both baroclinic and barotropic flows should exist in reality since an unstratified ocean is only a theoretical idealisation. For example, tidally forced internal gravity waves may be directly excited by eccentricity tidal forces in Enceladus, as proposed by Tyler (2011) in his stratified ocean scenario, which results in an increased tidal heating. They also contribute to enhance the tidal response of global ocean planets (Auclair-Desrotour et al. 2018).

**Fig. 8:**
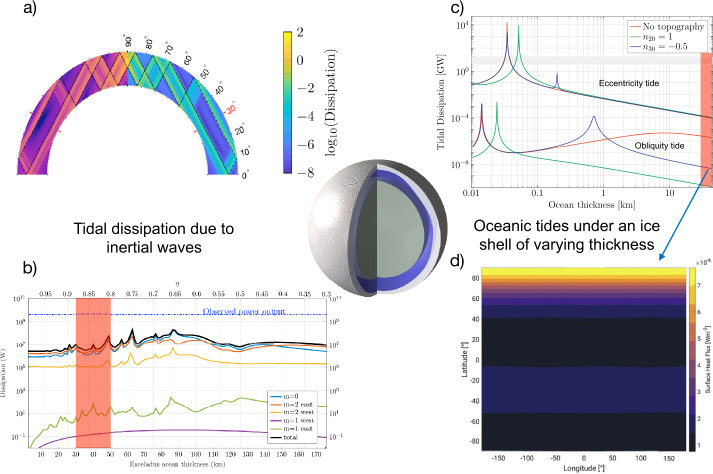
a) Viscous dissipation amplitude due to tidally-excited inertial waves obtained for Ekman number of $10^{-9}$ and inner to outer ocean radius ratio of 0.73 (considered as roughly representative of Enceladus’ ocean) and b) corresponding total dissipated as a function of ocean thickness (adapted from Rovira-Navarro et al. 2019). c) Corresponding tidal dissipation as a function of ocean thickness between models with an ocean of constant thickness and oceans with degree two and three thickness topography, assuming either eccentricity and obliquity tides, and d) corresponding time-averaged tidal dissipation patterns for a case with the obliquity tide case and an ocean with degree three topography (adapted from Rovira-Navarro et al. 2020). The pink band on panel b and c represents the estimated range of ocean thickness

The inertial (or gyroscopic) waves (e.g. Gerkema and Zimmerman 2008) are another – though secondary – type of internal waves that may be tidally excited in the oceans of icy moons (e.g. Rekier et al. 2019; Rovira-Navarro et al. 2019). These waves are restored by the Coriolis force, and thus develop in unstratified fluid layers. In the case where the ocean is stably stratified, inertial waves are mixed with gravity waves, which yields the so-called internal inertial-gravity waves (e.g. Gerkema and Zimmerman 2008). These waves are suspected to play an important role for energy dissipation in giant planets and stars (e.g. Ogilvie and Lin 2004; Ogilvie 2009, 2013; Rieutord and Valdettaro 2010; Lainey et al. 2017). For subsurface oceans, there are still a lot of debates and unknowns regarding their dynamical regimes (Soderlund 2019; Amit et al. 2020; Ashkenazy and Tziperman 2021; Bire et al. 2022; Kvorka and Čadek 2022; Soderlund et al. 2024) and the degree of oceanic stratification (e.g. Lobo et al. 2021; Kang et al. 2022b). Although the hydrostatic and traditional approximations used in the shallow water equations properly account for the predominating forced surface modes, they preclude the propagation of inertial modes. The latter are therefore studied in specific frameworks that relax these approximations by including, for instance, the full Coriolis force (non-traditional approximation; see e.g. Tort et al. 2014). By considering subsurface oceans of homogeneous densities for Europa and Enceladus, Rovira-Navarro et al. (2019) show that the inertial waves that could be indirectly forced by the equilibrium tide would lead to internal tidal flows of significant amplitudes (Fig. 8 a, c). Nevertheless, they note that the resulting tidally dissipated energy still remains several orders of magnitude smaller than Europa’s radiogenic heating and Enceladus’ observed heat flux (Fig. 8 c). Similar conclusions are drawn by Rekier et al. (2019) from the study of libration-induced inertial waves in Enceladus’ ocean. More recently, Idini and Nimmo (2024) studied inertial waves in a stably-stratifed ocean in the context of Titan, and showed that resonant stratification may significantly amplify the tidal response.

#### Tidal Dissipation Mechanisms

Analogous to solid layers, the tidal response of fluid layers is accompanied with energy dissipation. In oceans, this energy dissipation is mainly caused by the friction of the barotropic tidal flows against the oceanic floor and the overlying ice shell. However the term ‘friction’ should be taken here in a general sense, as it refers to several dissipative mechanisms. Even though the configuration of subsurface oceans is significantly different the Earth’s ocean, the Earth knowledge can provide useful physical constraints on the dissipative processes. On the Earth, tidal flows are primarily damped by the bottom drag due to turbulent friction in shallow seas. The work done by bottom drag is proportional to cubed velocity. As a consequence, it gets negligible in deep oceans where the tidal flows remain small compared to shallow seas/thin oceans.

Energy dissipation in the deep ocean is actually caused by another kind of friction that is directly related to the stable stratification of the water column. Through their interactions with the small scale topographic features of the oceanic floor, the long-wavelength barotropic tidal flows, excited by the forcing gravitational potential, are partly converted into gravity waves. These waves act as an energy sink for the oceanic tide since they remove kinetic energy from the tidal dynamics, this energy being eventually dissipated by turbulent and viscous frictions (e.g. Gerkema and Zimmerman 2008). This mechanism enters into the momentum equation as an additional linear drag, called ‘Rayleigh drag’ (e.g. Jayne and St. Laurent 2001; Tyler 2011; Matsuyama 2014; Auclair-Desrotour et al. 2018; Rovira-Navarro et al. 2023).

It is noteworthy that, in the linear theory, bottom drag and the drag of internal waves are both described by an effective Rayleigh drag parametrised by the so-called Rayleigh drag frequency (or coefficient), $\sigma _{\mathrm{R}}$, and formulated as $\boldsymbol{f}_{\mathrm{R}} = H \sigma _{\mathrm{R}} \boldsymbol{u}$. While this effective Rayleigh drag is estimated to be around $\sigma _{\mathrm{R}} \sim 10^{-5}~{s^{-1}}$ for Earth (e.g. Webb 1980; Egbert and Ray 2001; Jayne and St. Laurent 2001; Farhat et al. 2022), no constraints exist for subsurface satellites, which allows a large range of values to be used (typically, $\sigma _{\mathrm {R}} {\sim } 10^{-11}{-}10^{-5}~{\mathrm {s^{-1}}}$; see e.g. Matsuyama et al. 2018). Note that, as discussed by Matsuyama et al. (2018), the Rayleigh drag, $\boldsymbol{f}_{\mathrm{R}}$, can be used to mimic the dissipation generated by bottom friction, $\boldsymbol{f}_{\mathrm{B}}$. These authors established a relationship between the dimensionless drag coefficient of the non-linear term describing bottom friction, $C_{\mathrm{d}}$, and the Rayleigh drag coefficient, $\sigma _{\mathrm{R}}$, by assuming that the two drags generate the same energy flux for the same horizontal velocity field. Finally, one should pay attention to the fact that the bottom and Rayleigh drag, $\boldsymbol{f}_{\mathrm{B}}$ and $\boldsymbol{f}_{\mathrm{R}}$, are both divided by the ocean depth when included in the shallow-water momentum equation given by Eq. (24), given that the drag is averaged over the water column in this equation (see e.g. Matsuyama et al. 2018; Motoyama et al. 2020).

### Dynamical and Thermal Implications

Combined with the wave resonances detailed in Sect. 5.2, the above dissipative mechanisms have strong repercussions on the thermal-orbital evolution of planet-satellite systems, as they usually result in increased tidal heating and energy dissipation. The Earth’s oceanic tides are typically responsible for nearly ${\sim} 90{-}95\%$ of the tidally dissipated energy (e.g. Egbert and Ray 2001), meaning that they primarily drive the long-term evolution of the Earth-Moon distance and Earth length-of-day (e.g. Webb 1982; Daher et al. 2021; Tyler 2021; Farhat et al. 2022). Besides, their dependence upon the forcing frequency is consistent with the past variations of energy dissipation that can be inferred from geological data, which show that the gravitational tidal torque exerted on Earth during the Precambrian (${\sim} 1.13 \times 10^{16}$ J) was roughly a quarter of the present-day torque (${\sim} 4.51 \times 10^{16}$ J) in magnitude (e.g. Zahnle and Walker 1987; Farhat et al. 2022). In particular, the resonances of the predominant gravity modes markedly alter the climato-orbital history of the Earth-Moon system reconstructed in the recent solutions (e.g. Daher et al. 2021; Tyler 2021; Farhat et al. 2022).

Such resonant amplifications are likely in the icy satellites of the outer Solar system. Considering Europa, Tyler found that resonantly excited Rossby waves due to obliquity forcing could lead to tidal flows with a kinetic energy 2000 times larger than that of the flow generated by the dominant tidal forces (Tyler 2008). This first analysis by Tyler (2008), however, neglected the effect of dissipation which should damp the resonant flow and assumed a relatively high obliguity (0.05^∘^). He also suggested that Enceladus’ extreme heat flux could be explained by strong obliquity-forced tidal flows in its subsurface ocean if Enceladus’ spin axis is tilted with respect to its orbital plane by at least $0.05^{\circ}$ (Tyler 2009, 2011). The latter scenario, however, is discounted by Chen and Nimmo (2011) and Chen et al. (2014), who argue that Enceladus’ obliquity should be actually less than $0.0015^{\circ}$, and that important ocean tidal heating is unlikely for most satellites of the outer Solar system. Several authors invoke the possibility of strong resonantly excited eccentricity tides, but only if the subsurface ocean is ${\sim}1$ km thick or thinner (e.g. Matsuyama 2014; Tyler 2014; Kamata et al. 2015). Even though such a strong dissipation may have occurred during some specific periods, a thin ($\le 1$ km) ocean on Enceladus is clearly inconsistent with the existing topography, gravity and rotation data collected by Cassini, which rather indicate a thick ocean, potentially as thick as 50-60 km in the south polar region (Čadek et al. 2019; Hemingway and Mittal 2019). Moreover, the strong damping effect of the overlying ice crust may further reduce the resonant response and associated dissipation (Beuthe 2016; Aygün and Čadek 2023).

Beyond these considerations on the possible present states of liquid water reservoirs within icy moons, it is worth bearing in mind the full dynamical process leading to such states. In particular, a liquid ocean attempting to freeze is likely to experience strong tidal heating during its evolution since it must pass through resonant configurations, which may even lead to an equilibrium state if the produced heat is sufficient to melt the lower bound of the ice crust (e.g. Tyler 2011). Also, as the above studies mostly rely on the shallow water approach, alternate scenarios take into account the heating induced by internal waves. The studies exploring these scenarios, which mainly focus on Enceladus, show that the energy dissipated by tidally forced gravity waves in stably stratified oceans may exceed that resulting from surface waves (e.g. Tyler 2011; Rovira-Navarro et al. 2023), while the resonant excitation of inertial waves can significantly increase the heat production (e.g. Rekier et al. 2019; Rovira-Navarro et al. 2019).

Subsurface water oceans are not the only fluid layers playing a role in the evolution of rocky bodies. Magma oceans, namely liquid layers of melted rocks, are expected to affect this evolution as well. Typically, the strong tidal heating occurring in the primitive Earth and lunar surface magma oceans of the Hadean had repercussions on the early dynamical and thermal evolution of the Earth-Moon system (e.g. Chen and Nimmo 2016). This tidal heating was, for instance, capable of prolonging the duration of the lunar magma ocean for ${\sim} 200{-}300$ Myr (e.g. Meyer et al. 2010), and it may have partly controlled the crystallisation process in the Earth’s solidifying crust through thermal convection (e.g. Monteux et al. 2023). Similarly, Tyler et al. (2015) show that the region of the parameter space where the tidal heat released in Io’s magma ocean reaches the observed heat flux is wider when the ocean is treated as a fluid than when it is treated as a solid.

Furthermore, the increased energy dissipation induced by the dynamical tide allows subdominant tidal forces to generate heat flows exceeding those associated with the predominant forces for specific resonant configurations. With this effect in mind, Hay et al. (2020) investigate the possibility that Moon-Moon tides – namely the tides raised on moons by other moons – can lead to substantial energy dissipation. Although they could be in theory detectable on Ganymede by Juice’s 3GM experiment (De Marchi et al. 2022), Moon-Moon tides are much weaker than the tides raised by the planet because of the great difference in mass of the perturbers. Therefore, their contribution to the tidal energy budget is negligible in the general case. Hay et al. (2020) argue, however, that this contribution can affect the total energy budget for very specific ocean thicknesses if the ocean is inviscid enough to build a strongly resonant behaviour, which corresponds to $\sigma _{\mathrm{R}} < 10^{-6}~{\mathrm{s^{-1}}}$, typically. While this value of the drag frequency is less than the Earth value by one order of magnitude, Matsuyama et al. (2018) suggest that it is not unlikely regarding the icy moons of the outer Solar system, as $\sigma _{\mathrm{R}}$ can reach values as low as $\sigma _{\mathrm{R}} \sim 10^{-11}~{\mathrm{s^{-1}}}$ in their estimates. Nevertheless, one should bear in mind that there are no constraints for the bottom drag coefficients in icy satellites. Such extreme values should thus be considered as theoretical lower limits rather than nominal values.

### Coupling with the Overlying Ice Shell and the Solid Part

Contrary to present-day Earth’s oceans, liquid water layers in icy satellites are overlaid by ice crusts whose thickness depends on the thermal-orbital history of the bodies. By constraining the thickness of Europa’s ice shell from numerical experiments of thermal convection, Tobie et al. (2003) found it to be about $20{-}25$ km, with lateral variations of about 5 km. Such constraints can also be obtained from the measurements of tidal gravity fields and vertical displacements, although uncertainties in the shear modulus feed directly into uncertainties in the ice shell thickness (e.g. Wahr et al. 2006; De Marchi et al. 2022). The coupling of the ocean with the overlying shell was first examined in the framework of the viscoelastic-gravitational theory, where all inertial effects are ignored (e.g. Tobie et al. 2003; Beuthe 2015a). This approach shows that the tidal response of the crust is controlled by its membrane spring constant and its effective Poisson’s ratio, which quantify the elasticity and compressibility of the material, respectively (e.g. Beuthe 2015a). However, it does not capture the coupling of the oceanic tidal modes with the shell.

In order to remedy these limitations, several authors adopted a more sophisticated formalism by self-consistently including the ocean-crust interactions into the LTEs (e.g. Beuthe 2016, 2018, 2019; Matsuyama et al. 2018). These models are based upon the membrane theory, where the shell is assumed to be thin compared with the radius of the body (e.g. Beuthe 2008, 2018). They predict that the crustal constraint strongly attenuates the resonances of barotropic modes, thus reducing the associated oceanic energy dissipation (see e.g. Beuthe 2016). This damping effect is expected to be more pronounced on Enceladus than on Europa as it is straightforwardly related to the effective elasticity of the overlying shell, which increases as the size of the body decreases (Matsuyama et al. 2018).

Furthermore, it is noteworthy that the thickness variations of the shell play an important role as well, as the crustal thinning of Enceladus at the south pole can amplify the local tidal stresses by one order of magnitude (Beuthe 2018). Conversely, the horizontal thickness variations of the ice shell are themselves dependent upon the oceanic tidal heat flow, which is not homogeneous over the sphere (e.g. Matsuyama et al. 2018). Rovira-Navarro et al. (2020) investigated how degree-two and degree-three ice/ocean interface topography could affect the response of Enceladus’s ocean to eccentricity and obliquity tides (Fig. 8 c and d). These results show that the ice shell thickness variations shift the resonant peak to different values of ocean thickness. However, for ocean thicknesses representative of present-day Enceladus, the produced total power remains unchanged for eccentricity tides and is reduced for obliquity tides when varying thickness is taken into account. Even though ice shell thinning should result in a higher dissipation rate at the south pole for obliquity tides, the produced heat flow from the ocean remains several orders of magnitude below the observed values. The predicted heat flow remains below $10^{-2}$ mW m^−2^ while the observed heat flow exceeds 100 mW m^−2^ (Spencer et al. 2013, 2018).

Last but not least, the solid part of an icy moon is not rigid, as described in simplified idealisations (e.g. Longuet-Higgins 1968). Instead, it responds visco-elastically to the surface loading and self-attraction variations of the ocean (e.g. Peltier 1974). To refine the Earth tidal theory, Hendershott (1972) introduced the deformations of solid regions in the LTEs that describe the global ocean tides, thus showing that the solid-ocean coupling leads to more excited harmonics. This effect is due to the differences between the spherical harmonics and the Hough functions featuring the horizontal structures of the tidally induced barotropic modes, the two sets of eigenfunctions being equivalent in the static limit solely (e.g. Lee and Saio 1997). In the context of icy moons, models including the deformations of solid regions and self-attraction variations show that the visco-elastic adjustment of the solid part slightly attenuates the resonant peaks of the dynamical tide. These peaks are, moreover, shifted by the solid response, which can cause orders of magnitude changes in the dissipated energy flux if the oceanic configuration is close to a resonance (e.g. Matsuyama 2014). By solving the three-dimensional Navier-Stokes equations taking into account viscoelastic coupling with the overlying ice shell and underlying rock layer, Aygün and Čadek (2023) show that the resonant modes both in terms of peak amplitude and frequency can be significantly modified, resulting in a dissipation power much smaller than what is predicted using the standard LTE approach.

## Tidal Response of Icy Shells: Implications for Surface Activity

### Tidally-Induced Melting in Convective Ice Shells

Depending mainly on the ice shell thickness and ice viscosity, the heat released from the moon’s interior can be transferred through an ice shell by either conduction or convection. As tidal heating is strongly dependent on the viscosity with optimal dissipation for the viscosity of the order of $10^{13}-10^{15}$ Pa s (see Sect. 3, Fig. 4), heat transfer and heat production by viscous friction are strongly coupled, in particular for convective ice shells. In a conductive ice shell, the heat transfer is very inefficient and the thermal profile is characterized by an almost linear increase of temperature with depth, resulting in only a small warm portion of the ice shell just above the ice/ocean interface, where viscosity is close to the optimal value for viscous dissipation. On the contrary, in a convective shell, the heat transfer is much more efficient and the thermal structure is characterized by a warm, nearly isothermal sublayer below a cold, highly-viscous conductive lid. The relative thickness of the convective and conductive sublayers depends on various factors, such as ice viscosity, amount of tidal heating, mobility/deformation of the lid etc. Consequently, tidal heating can vary by orders of magnitude between conductive and convective cases, as the thickness of the dissipative ice can vary from less than 1 km for a thin conductive layer to several tens of kilometers for a convective ice layer. Moreover, as viscosity varies laterally in a convective layer, tidal heating can be locally increased in regions where the local viscosity is equal to the optimal viscosity value for dissipation (Tobie et al. 2003). For optimal viscosity equal to the viscosity at the melting point, tidal heating is maximized at the hot thermal boundary layer which can favor the initiation of warm upwelling plumes (Běhounková et al. 2013).

**Fig. 9:**
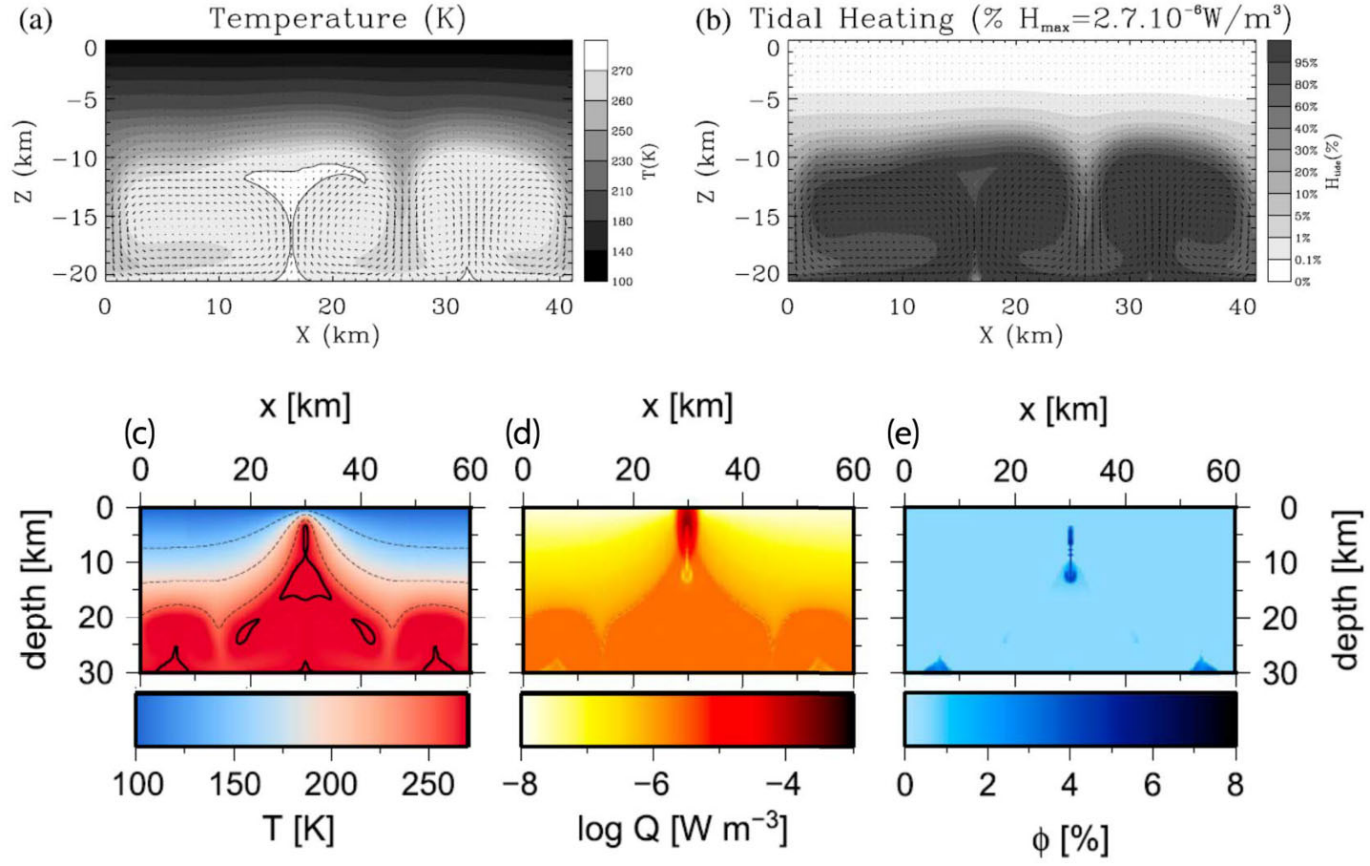
*Top*: Temperature (a) and volumetric tidal heating rate (b) in a convecting ice shell of Europa with viscosity-dependent volumetric heating (assuming a Maxwell model) (Tobie et al. 2003). The black line in panel (a) marks the area where temperature equals the melting temperature of pure water ice. *Bottom*: Temperature (c), heating rate (d), and porosity (e) in Europa’s ice shell with fixed frictional heating at the fault and viscosity-dependent volumetric heating (assuming an Andrade model) (Kalousová et al. 2016). Dashed contours in panel (c) mark temperatures of 150, 200, and 250 K, the thick contour denotes the melting temperature (270 K)

Focusing tidal heating in warm upwelling plumes can lead to strong melting (Tobie et al. 2003), potentially resulting in surface disruptions due to melt drainage and collapse, proposed as a possible origin of chaotic terrains on Europa (Sotin et al. 2002) and for initiating the activity on Enceladus during periods of enhanced eccentricity (Běhounková et al. 2012). Accumulation of melts in tidally-heated plumes is expected to affect their dynamics owing to the density difference between the generated meltwater and the ice matrix. The produced meltwater represents a new source of buoyancy that can act to strengthen or weaken the thermal convection and should therefore be considered in the convection model. Solving the full problem of two-phase convection is numerically demanding and thus various approximations have been considered. At first order, one can neglect the differential motion between the two phases and assume that water is simply advected by the solid ice matrix. As illustrated in Fig. 9, melt produced by enhanced tidal heating in convective plumes (top) or at the base of active strike-slip faults (bottom) in the context of Europa results in a downward motion of the partially molten ice due to negative buoyancy. The melt volume and associated dissipation rate tend to increase during this downward migration which amplifies this effect. As shown by Tobie et al. (2003), the coupling between tidal heating and melt production leads to strong time variations in convective dynamics with intermittent periods of thermal plume growth followed by periods of melt-induced plume collapses. This results in significant variations in heat flux variations at the ice/ocean interface, with important consequences for mass and chemical exchanges between the ocean and overlying ice shell. Such downward motions of partially molten ice could entrain any salt and non-ice constituent present in the ice shell to the ocean and such cold salty water may significantly affect the dynamics of the underlying ocean.

More recent studies take into account the differential motion between the two phases (Kalousová et al. 2018; Kalousová and Sotin 2018, 2020). In such cases, the water velocity can be up to a few orders of magnitude larger than the ice velocity, depending mostly on the amount of available water, the ice permeability, the liquid water viscosity, and the convection regime (stationary or chaotic). This approach was used for the deep ice layers that are predicted in large moons such as Ganymede or Titan. These layers are made of high-pressure ice polymorphs, which are denser than liquid water at the corresponding pressures (hundreds of MPa to units of GPa). In that setting, the phase-change-induced buoyancy acts in the same direction as the thermal buoyancy and thus enhances the convective motions. In a pure ice setting (i.e. without the addition of salts or other non-ice compounds), a so-called temperate layer (where ice is at the melting temperature) is established at the boundary between the convecting high-pressure ice and the above-lying liquid water ocean. This temperate layer effectively eliminates the top thermal boundary layer and thus the convective pattern can be described as active upwelling plumes (that enable the transport of liquid water) and passive background downwelling (Kalousová et al. 2018; Kalousová and Sotin 2018, 2020). This process is similar to silicate melts ascending through silicate mantles (e.g. Ogawa and Yanagisawa 2011; Ogawa 2014). Even though the tidal dissipation is expected to be two orders of magnitude smaller in the high-pressure ice layer compared to the ice I layer above the liquid ocean, tidal heating during periods of enhanced eccentricity could significantly affect the heat budget in the high-pressure ice layer. Consequently, the melt production in the high-pressure ice layer would increase, thus favoring material exchange between the underlying silicate core and the overlying ocean.

### Tidal Deformation and Heating Along Tectonic Faults

Tidal deformation and the associated dissipative heating within planetary ice shells are expected to be intensified by the presence of active faults or fault systems within these shells. Currently, the system consisting of four prominent faults located in the southern polar region of Enceladus, commonly referred to as “tiger stripes”, stands as a preeminent exemplar of such structures among the known ocean worlds. Instruments aboard the Cassini spacecraft observed remarkable activity in the form of jets of vapor and ice particles emanating from these south polar fissures (Porco et al. 2006; Postberg et al. 2018). The modulation of brightness, reflecting the solid particles content, on the tidal timescale (e.g., Hedman et al. 2013; Nimmo et al. 2014; Ingersoll et al. 2020) implies tidal control of the process (Hurford et al. 2007). However, the exact interpretation of this activity signal has not been straightforward due to the more than five-hour observed lag between the timing of the predicted extensional stress maximum and the main activity peak (Nimmo et al. 2014). Several attempted explanations, including viscoelasticity (Nimmo et al. 2014; Běhounková et al. 2015), libration (Nimmo et al. 2014), or local hydraulic processes (Kite and Rubin 2016), have either been disproved, doubted, or raised further questions. Additionally, the measurements seem to indicate either weaker or absent modulation of the vapor mass flux on the tidal time scale (Hansen et al. 2020) with potential changes on shorter (hour) time scale (Denny et al. 2024).

Pleiner Sládková et al. (2021) and Berne et al. (2024) observed that the two maxima of jets’ activity can be correlated with modeled strike-slip motions rather than normal stresses or displacements at the faults. Souček et al. (2024) proposed a model providing a physical explanation for this correlation, combining a global shell deformation model with detailed modeling of water and vapor transport processes within the partially flooded fissures, in line with models by Kite and Rubin (2016), and Nakajima and Ingersoll (2016). These interpretations thus support the view of tiger stripes as strike-slip faults penetrating the entire shell thickness, whose activity is maintained by incessant tidal forcing.

**Fig. 10:**
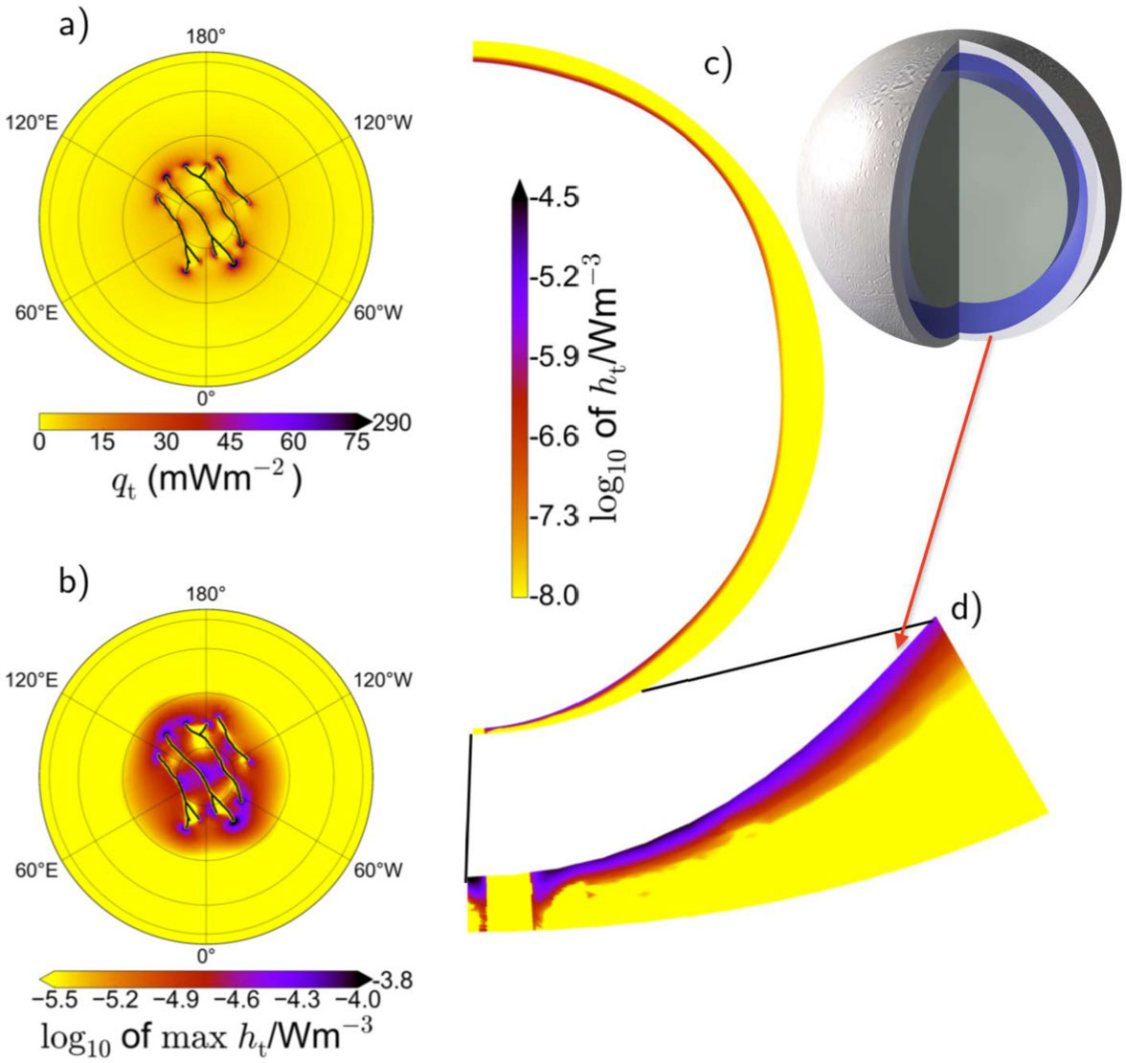
Tidal dissipation in the SPR of Enceladus (computed for pseudo-Andrade rheological model and reference viscosity at the melting point $\eta _{0} = 3\cdot 10^{14}$ Pa s). Panels show a) Depth-integrated heating power expressed as equivalent tidal heat flux in the southern hemisphere. b) Maximum tidal heating rate with respect to depth. c) Tidal heating rate plotted at meridional cross-section $\phi{=}0^{\circ}$. d) Tidal heating rate near the south pole. The thickness of the ice shell in panel d is exaggerated by a factor of 3. The faults in this study are considered to be frictionless, consequently, the tidal heating is zero inside fault zones. The SPR (panels a and b) is shown in stereographic projection for latitudes 60^∘^–90^∘^ S. Adapted from Souček et al. (2019)

The effect of the presence of faults on the global tidal deformation of Enceladus has been investigated by means of numerical models of the tidal deformation in the studies by Souček et al. (2016), Běhounková et al. (2017), Souček et al. (2019), Pleiner Sládková et al. (2021), and its validity was corroborated by subsequent research efforts by Berne et al. (2023). It has been demonstrated that the thinning of the southern polar shell, in conjunction with the existence of these faults, synergistically culminates in a substantial amplification of tidal deformations, particularly when these faults are treated as frictionless slots. Consequently, this leads to a localized augmentation of dissipation in the proximity of the fault zones, particularly at the tips of these faults (Fig. 10). The cumulative impact of the presence of faults on the dissipative heat budget of the southern polar region of Enceladus has been, under the above assumptions, estimated to be of the order of units of GW (Souček et al. 2019). Owing to the local effects, especially at the fault tips, it is conceivable that these effects could be responsible for generating local thermal heat flux or temperature anomalies such as the ones inferred by Le Gall et al. (2017). Even if tidal dissipation can be locally enhanced, the total power generated along such active faults estimated to be less than 2.1 GW (Souček et al. 2019) remains too small to explain the total heat budget of Enceladus estimated to 25-30 GW (Čadek et al. 2016; Choblet et al. 2017).

The phenomenon of friction occurring at the faults (neglected in Souček et al. 2016; Běhounková et al. 2017; Souček et al. 2019) introduces an additional source of dissipative heating (Pleiner Sládková et al. 2021). Simultaneously, it results in the damping of deformation (Pleiner Sládková et al. 2021; Berne et al. 2023), and consequently, a decrease of volumetric dissipation in the vicinity of the faults. The combined impact of these processes on the dissipative heat budget within the fault’s immediate surroundings varies depending on the nature of the frictional interaction and can either amplify or reduce the overall dissipative heat budget in the fault’s neighborhood. In the case of a simplified Coulomb-type friction model, the frictional contribution to the comprehensive dissipative heat budget of Enceladus’s South Polar region has been estimated to be less than 1 GW.

As demonstrated by Pleiner Sládková et al. (2021), the inclusion of friction within a tidal deformation model applied to Enceladus’s icy shell has unveiled an intriguing and unexpected dynamic effect, potentially carrying implications for local tectonics and surface morphology in the southern polar region. The effect stems from the inherent asymmetry of Coulomb-type friction, characterized by weaker frictional contact during the extensional phase of the tidal period and stronger contact during the compressive phase. Consequently, an initially hydrostatically pre-stressed shell with embedded Coulomb-type faults would reach after some time a novel dynamic “equilibrium” wherein the average stress conditions at the faults transition towards a more compressive state. This dynamic adjustment results in the development of predominantly compressive background stress within the SPR of Enceladus, reaching a magnitude comparable to that of the dynamic tidal stresses itself (${\sim}100$ kPa). The possible implications of this background stress field have not been studied yet.

**Fig. 11:**
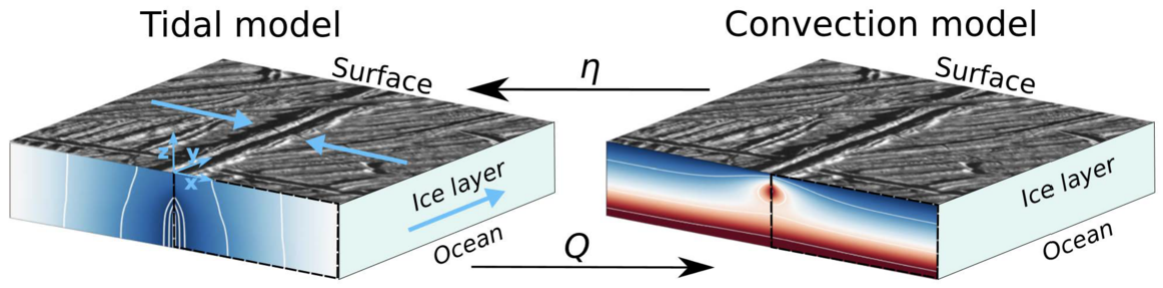
Two-component model of tidal walking process at the strike-slip faults on Europa. The tidal model (left) solves for the (viscoelastic) strike-slip motions due to diurnal tides and the (viscous) convection model (right) computes the long-term thermal evolution. The coupling between the models occurs through the tidal heating rate and the viscosity. From Sládková et al. (2020)

In studies conducted by Preblich et al. (2007) and Sládková et al. (2020), a similar mechanism involving Coulomb-type friction acting on tidally loaded faults in a viscoelastic shell was demonstrated to offer a self-consistent explanation for the concept of “tidal walking” (Fig. 11). This conceptual idea has been proposed by Hoppa et al. (1999) to account for observed lateral offsets on numerous lineaments interpreted as strike-slip faults on the surface of Europa (Schenk and McKinnon 1989). Through direct numerical modeling, it was confirmed that the asymmetry in frictional forces, in conjunction with viscoelastic relaxation occurring within the bulk material surrounding these faults, has the potential, under specific favorable conditions, to result in the gradual accumulation of irreversible slip along the faults. According to Sládková et al. (2020), the favorable conditions in question necessitate the fault’s penetration from the surface either all the way to the internal ocean or to a sufficiently low-viscosity zone within the moon’s icy shell. It was found that such conditions are unlikely under the present-day conditions on Europa and the present magnitude of tidal forcing. However, it cannot be ruled out that these conditions may have been realized during Europa’s past history, particularly during periods of higher orbital eccentricity and, consequently, greater tidal forcing.

In summary, considering both the local and regional effects associated with the mechanical and thermal responses of faults within the outer shells of icy moons is undeniably crucial for obtaining a comprehensive understanding of their tidal behavior. Unfortunately, due to the substantial computational challenges involved in this type of modeling, there are currently only a limited number of studies that have addressed this complex phenomenon.

### Tidal Dynamics in Water-Filled Cracks

Another notable instance of a tidally induced and highly localized process with significant potential implications for local thermal conditions is the phenomenon of tidal water plumbing within faults that traverse the entire shell, from the surface down to the internal ocean. This process involves periodic transitions between compressional and extensional conditions within a water-filled fault system, resulting in dynamic changes in the elevation of the water column. The movement of liquid water under idealized conditions, akin to those found in the Enceladean tiger stripe system, was investigated by Kite and Rubin (2016). Their research demonstrated that mechanical dissipation due to turbulent flow occurring within the water channel system itself can, under specific favorable circumstances, account for a significant portion (units of GW) of the endogenic heat flux ${\sim}5$ GW from the tiger stripes (Spencer et al. 2013). It is important to note that the authors considered a relatively thick shell in the SPR (30 ± 10 km), while more recent estimates (Beuthe et al. 2016; Čadek et al. 2016, 2019; Hemingway and Mittal 2019) suggest thinner shells (only a few km in the SPR). This would accordingly reduce the estimated heating contribution. Nevertheless, this mechanism remains a plausible component of the puzzle concerning the heat budget of the Enceladean SPR.

The water plumbing system proposed by Kite and Rubin (2016) assumed a direct communication with the underlying ocean and idealized geometry. In reality, the water system may be more tortuous and characterized by local enrichment in meltwater. A favorable “resonant” state, with peaking hydraulic dissipation, is more difficult to reach in the case of more complex geometry. However, even if a resonant state is not reached, the presence of meltwater, potentially produced locally under the action of enhanced tidal heating (Nimmo et al. 2007) or injected from the underlying ocean, may influence the mechanical properties of the crack system and thus further amplify tidal motions.

## Geophysical Constraints on Tidal Processes by Future Exploration Missions

Tidal deformation and associated dissipation can be assessed using accurate gravimetric and altimetric measurements, as well as thermal emission in case of very strong and localized dissipation. So far, Titan is the only moon for which tidal response has been directly measured from the accurate determination of the time-varying gravity field (Iess et al. 2012; Durante et al. 2019). The inferred potential Love number, $k_{2}= 0.616 \pm 0.067$, implies that Titan possesses an internal ocean under an ice shell whose thickness remains poorly constrained. As the tidal response depends on both the ice shell thickness and ocean density (Mitri et al. 2014) as well as possibly on the degree of ocean stratification (Idini and Nimmo 2024), it is only possible to provide a broad range of possible ice shell thickness ($\sim 50-100$ km) and ocean density (1150-1300 kg m^−3^). More recently, Goossens et al. (2024) re-evaluated the Love number to about 0.375, suggesting an ocean with a low density and under a thick ice shell. Future measurements are needed to be conclusive. Notably Dragonfly may provide additional constraints by monitoring very accurately the atmospheric pressure variations due to the gravitational tides (Charnay et al. 2022).

For the Galilean moons, in particular Ganymede and Europa, the precision of the observations to be performed by the ESA JUpiter ICy moons Explorer (Juice) mission (Grasset et al. 2013) and the NASA Europa Clipper mission (Howell and Pappalardo 2020) will be much better. The two missions will measure both the time-varying gravity field and surface deflection using radio tracking (Cappuccio et al. 2020; Mazarico et al. 2023) and altimetry, with laser technique for Juice (Steinbrügge et al. 2015) and radar technique for Europa Clipper (Steinbrügge et al. 2018). Europa Clipper will measure the tidal distortion by performing about 50 close flybys as low as 25 km (Roberts et al. 2023), while Juice will monitor the tidal response during a dedicated geophysical campaign on a low-altitude circular orbit (of at least 130 days at 500 km and possibly at 200 km during the extended mission) (Van Hoolst et al. 2024). The expected accuracy for the gravitational Love number, $k_{2}$, is $\le 0.02$ for Europa Clipper (Mazarico et al. 2023) and $\le 0.0001$ for Juice (Cappuccio et al. 2020). In the case of Ganymede, the imaginary part of $k_{2}$ will also be determined with a comparable accuracy by Juice, while for Europa it is still unclear if the precision will allow a quantification of the imaginary part of $k_{2}$ (Mazarico et al. 2023). Juice will also provide constraints on the gravitational Love number of Callisto with an accuracy comparable to Cassini at Titan (Cappuccio et al. 2022), which will permit the confirmation of the existence of a subsurface ocean.

The tidal response in terms of gravitational perturbations and surface deflections is at first order controlled by the ice shell thickness and the ocean density. The thermal structure of the ice shell which affects the viscosity and effective rigidity can also significantly affect the tidal response, as demonstrated in the case of Titan (Mitri et al. 2014). Even if the tidal response is measured very precisely, it is difficult to separate the effects of ice shell thickness, ocean density and thermal structure of the ice shell. By combining the Love numbers, $k_{2}$ and $h_{2}$, the effect of ocean density can be suppressed or at least significantly reduced, but still, the estimate of ice shell thickness will depend on the viscoelastic model assumption and on the assumed thermal profile in the ice shell. Additional geophysical measurements such as magnetic induction, radar sounding and gravity-topography ratio will be essential to constrain the ice shell thickness and the ocean properties (Van Hoolst et al. 2024; Roberts et al. 2023).

Owing to the very high precision of tidal monitoring by Juice at Ganymede, any subtle variation relative to the standard degree-two tidal response could be detected. For instance, Juice could detect even small changes in the pattern of tidal surface displacement and gravitational perturbations related to the non-zero obliquity of the satellite (Jara-Orué and Vermeersen 2016; Steinbrügge et al. 2023). Given the 1:1 spin-orbit resonance, the obliquity tides have the same period as the eccentricity tides and have to be considered in the determination of the Love numbers $h_{2}$ and $k_{2}$ from Juice data. Since both the orbital eccentricity and the obliquity can vary significantly (by 10% to almost 100%, Steinbrügge et al. 2023) over a few months, the amplitudes of the eccentricity and obliquity tides as well as their ratio will depend on time, and should be carefully monitored to correctly interpret the interior response. In addition to the main tidal interaction with Jupiter, the Galilean moons are subject to lower amplitude, time-dependent, tidal interactions with the other moons (Hay et al. 2020), resulting in multiple frequencies related to the Io-Europa-Ganymede-Callisto synodic frequencies. Even though much smaller than the main tidal response, these moon-moon tidal responses could be potentially detected at Ganymede using the 3GM radio science experiment onboard Juice (De Marchi et al. 2022), which may provide additional and independent constraints on the thickness of the ocean to within ∼10 km if one of the tides is resonant with a surface gravity wave of the ocean (De Marchi et al. 2022; Hay et al. 2022). Accurate tidal monitoring may also reveal lateral variations in ice shell thickness as well as the dynamical response of subsurface ocean and its coupling with the overlying ice shell (e.g. A et al. 2014; Beuthe 2016, 2018; Rovira-Navarro et al. 2023). Future modeling work dedicated to Ganymede taking into account the complexity of ocean-ice tidal coupling is required to anticipate the interpretation of the future very accurate data.

For Enceladus, the strong ice shell thickness variations and the fault system at the south pole make the tidal response much more complex (Běhounková et al. 2017; Souček et al. 2019; Marusiak et al. 2021; Berne et al. 2023). Detailed monitoring of tidal response would require an orbiter mission, similar to the GRAIL mission, performing dedicated gravity and altimetry mapping investigations (e.g. Ermakov et al. 2021; Genova et al. 2024). This would allow us to determine the time variations of the gravity field and surface deflection at degree two but also at higher degrees due to the complex response of Enceladus’ uneven ice shell. The effect of the unconsolidated weak core may be also revealed by an amplification of the degree-two pattern in the equatorial region (Marusiak et al. 2021), which would provide quantification of the amount of energy involved in hydrothermal activity. The global tidal monitoring would also be complemented by regional mapping of thermal emission and fault dynamics using InSar and radar-penetrating techniques in the south polar terrain (e.g. Choblet et al. 2021). Monitoring of plume activity during the tidal cycle and the correlation of individual jet activity with the fault dynamics would also provide insights into the eruption dynamics and the efficiency and mode of heat and material transport from the ocean to the surface, essential for selecting landing sites for future astrobiological investigations (e.g. Choblet et al. 2021; MacKenzie et al. 2021).

## Conclusion

In the present article, we have reviewed the different principles of tidal deformation and the mechanisms controlling the dissipation in icy worlds. With the exception of Io and Enceladus, we have no observational constraints on how much tidal heating is produced inside the interior of these moons. On Io, both thermal emission and astrometric data indicate a total dissipated power ranging between 65 and 125 TW (e.g. Spencer et al. 2000; Lainey et al. 2009). Such a strong dissipation and associated volcanic activity indicate a molten interior, however the depth and extent of the molten region still remains poorly constrained and debated (e.g. Hamilton et al. 2013; Tyler et al. 2015; Steinke et al. 2020; Kervazo et al. 2022; Davies et al. 2024). Detailed monitoring of Io’s tidal response by a future dedicated mission will be required to provide more constraining insights on the dissipation processes.

For Enceladus, the total heat emitted from the active south polar region is estimated between 4 and 19 GW (Spencer et al. 2006; Howett et al. 2011; Spencer et al. 2018; Nimmo et al. 2023), but the total heat budget should also account for heat lost by conductive cooling through the ice shell outside the south polar region, estimated to 25-30 GW (e.g. Čadek et al. 2016). This leads to a total power ranging between 30 and 50 GW, more than 100 times larger than the expected radiogenic power. Modeling of tidal dissipation in the ice shell including highly deformable faults (Souček et al. 2019) clearly indicates that ice dissipation in the active south polar region cannot account for more than a few Gigawatts. Turbulent dissipation in the water-filled cracks could potentially account for a few more gigawatts if a resonant mode could be sustained (Kite and Rubin 2016), but it still remains insufficient to counterbalance the huge heat loss ($> 25$ GW) required to sustain a global ocean. Strong viscous dissipation in the ice shell, as proposed by Hemingway and Mittal (2019), will require a low viscosity ($< 10^{13}-10^{14}$ Pa s) throughout the ice shell, which is clearly incompatible with the observed topography, requiring large variations in ice shell thickness and weak ice flow only possible for a high-viscosity conductive ice shell (Čadek et al. 2019).

For the present-day ocean/ice configuration as inferred from Cassini data, no oceanic dissipation model can explain the required heat production. Significant dissipation may occur only for thin ocean underneath a thick ice shell (e.g. Matsuyama 2014; Tyler 2014; Kamata et al. 2015), which may have occurred in the past, but which is far from the present-day configuration. The only identified dissipative mechanism that could produce at least 10 GW of power is dissipation in a highly deformable, weakly consolidated, water-filled porous rocky core (Choblet et al. 2017; Rovira-Navarro et al. 2022). The total power that could be produced is however highly dependent on the mechanical properties of such water-filled porous core, which remain poorly constrained. A variety of organic compounds and secondary minerals, such as clays, could significantly influence the dissipative properties of the core. It is likely that the chemical and mechanical properties of such a porous core evolve relatively rapidly on timescales of tens of million years owing to efficient water-rock alteration (e.g. Zandanel et al. 2021). So, even if strong dissipation in Saturn could maintain Enceladus in a dissipative state during long periods of time (Nimmo et al. 2023), it is unlikely that its interior properties remain constant over geological timescales.

The orbital configuration (eccentricity and semi-major axis) may have also significantly varied due to tidal expansion and orbital resonance encounters (e.g. Neveu and Rhoden 2019; Nimmo et al. 2023). Changes in orbital characteristics, interior structure and mechanical properties are strongly linked through tidal heating and tidally-induced water circulation. As shown recently by the evidence of a recently-formed ocean in Mimas (Lainey et al. 2024), the interior structure and total heat production can change on relatively brief periods of time ($<10$ Myr), making the coupled thermo-orbital modeling of such mid-sized moons very challenging. Once the orbital eccentricity reaches a critical value, such mid-sized icy moons can go in a few million years from a frozen state to a fully molten state with a thick global ocean circulating in a porous rock core. Oceanic dissipation may also play a role at the beginning of the ocean growth, accelerating the melting of the overlying ice shell. Once the eccentricity is damped, tidal heating ceases and the interior progressively freezes to the initial state. If the analysis of Lainey et al. (2024) is correct, Mimas should have experienced such a melting event a few million years ago and should go back to a more dormant state in the coming few million years. Several other moons, such as Saturn’s moon Dione or Uranus’ moons Ariel and Miranda may have experienced similar melting/freezing events, which could explain evidence for past high heat flows (White et al. 2017; Nimmo 2023). Whether Enceladus experienced several cycles of melting/freezing is still an open issue. The recent activation of Mimas’ internal dissipation suggests that, in the recent past, Enceladus may also have experienced a period with higher eccentricity and hence even higher heat production than at present (Noyelles et al. 2019). Future measurements including monitoring of degree-two tidal distortion as well as tidally-modulated deformation, eruption rate and thermal emission along the SPT faults will provide key information on the source of dissipation and how heat is released to the surface. Such measurements will require a dedicated polar orbiter. Future experimental works are required to quantify the dissipative function of weakly consolidated rocky materials and to better understand the role of low viscosity/fluid interstitial phase on the dissipation processes. A better understanding of the role of water in the mechanical properties of the fault zone is also required.

Europa has likely also experienced significant variations in ice shell thickness modulated by changes in eccentricity variations and hence in heat production (e.g. Hussmann and Spohn 2004). The geological record suggests that Europa should be now in a period of ice thickening (e.g. Figueredo and Greeley 2004; Doggett et al. 2009), even though the thickness and timing of the ice shell thickening is still uncertain. Depending on the ice shell thickness and eccentricity, different dissipation mechanisms may be predominant. During periods of a thin shell ($< 5-10$ km) and high eccentricity (at least twice the present-day value), dissipation along tectonic faults may be a dominant process, as the condition for propagating faults throughout the whole ice shell may be met, thus amplifying strike-slip motions (Sládková et al. 2020). Oceanic water injected in the cracks from the bottom of the ice shell may also add additional sources of dissipation if a resonant configuration similar to what has been proposed for Enceladus (Kite and Rubin 2016) is met. During periods of elevated eccentricity, tidal dissipation in the mantle may exceed the radiogenic power, thus resulting in internal melting and enhanced heat flux at high latitudes (Běhounková et al. 2021). This additional heat source favors the thinning of the ice shell, which should be even more pronounced in high latitudes under the joint contribution of dissipation in the mantle and in the ice shell. Ocean dissipation is not expected to play a key role during periods of thin shell/thick ocean.

During periods of thick shell and reduced eccentricity, dissipation is expected to be dominated by viscous dissipation in the convective part of the ice shell. For present-day eccentricity, such a dissipative process is expected to be sufficient to stop the ocean freezing when the ice shell thickness reaches a value of 20-40 km (e.g. Tobie et al. 2003; Howell 2021). During periods of lower eccentricity, much thicker ice shell may be reached, resulting in thin oceans potentially highly concentrated in salts. When such configuration is met, dissipation in the remaining oceanic layer could become a dominant process (e.g. Matsuyama 2014; Tyler 2014), preventing the ocean from full freezing even during periods of very low eccentricity. The amplitude and periodicity of eccentricity change on Europa are still poorly constrained. Modeling such eccentricity changes and the consequences on the ice shell state and thickness requires a better understanding of the coupling with Io and Ganymede via the Laplace resonance. Future measurements by Europa Clipper and Juice missions will provide some direct constraints on the strength of this coupling at present through astrometric measurements (e.g. Dirkx et al. 2017) as well as constraints on the tidal response of Europa (Mazarico et al. 2023; Steinbrügge et al. 2018), essential to determine its hydrosphere structure and to give insights on the dissipation processes. The interpretation of these measurements requires, however, experimental data on the viscoelastic response of ice and ice-salt mixture in the appropriate frequency regime.

For Ganymede and Callisto, tidal dissipation has a negligible contribution to their heat budget at present. However, tidal monitoring by the Juice mission will provide key constraints on their hydrosphere structure (Van Hoolst et al. 2024). For Callisto, the expected accuracy (Cappuccio et al. 2022) will not be sufficient to provide precise constraints on the ice shell thickness and ocean density, but it will be comparable to what has been achieved at Titan with Cassini (Durante et al. 2019). For Ganymede, very subtle time variations in the gravity field (Cappuccio et al. 2020) and surface deflections (Steinbrügge et al. 2015, 2023) will be measured, offering unprecedented insights on the tidal response of an ice-covered ocean world. Such measurements will potentially reveal resonant modes in the ocean excited by moon-moon interactions (De Marchi et al. 2022) or related to ocean stratification (e.g. Idini and Nimmo 2024). Future model developments are needed to carefully predict the oceanic response and its coupling with the overlying ice shell and deep structure. Even if tidal heating is negligible at present, it might have significantly contributed in the past during the initial tidal despinning to their tidally-locked spin-orbit resonances (Tobie et al. 2014; Journaux et al. 2020) and during periods of elevated eccentricities (Bland et al. 2009). The tidal despinning which should occur on brief periods of time shortly after accretion could potentially rise the internal temperature by 25-50 K (Tobie et al. 2014), thus favoring the initiation of ice melting in the undifferentiated proto-core. Depending on how Ganymede enters the Laplace resonance, it may have experienced prolonged periods of elevated eccentricity (up to 10 times the current value), leading to tidally dissipated power of the order of 1 TW comparable to the radiogenic power (Bland et al. 2009). Geological and compositional mapping by Juice will provide insights into the change in tectonic regime and crater relaxation, thus constraining any past heat flux and changes in ice shell thickness associated with enhanced tidal heating.

On Titan, the contribution of tidal heating at present is more uncertain. It has long been assumed that tidal dissipation should be small at present in order to explain its elevated eccentricity (Sagan and Dermott 1982; Sears 1995; Sohl et al. 1995; Tobie et al. 2005b). The indication of rapid orbital expansion of Titan due to strong dissipation in Saturn (Lainey et al. 2020) suggests that its eccentricity may decay more slowly that previously estimated. In this case, tidal heating may still be a significant, if not dominant, source of internal heating. Even though Titan is the first icy moon for which the tidal Love number, $k_{2}$, has been inferred from gravity measurements (Iess et al. 2012; Durante et al. 2019), little is known on its tidal response as the measurement uncertainty still remains too large to be conclusive. The apparently elevated Love number could be explained either a moderately to very salty ocean (Mitri et al. 2014), or even by a low-density ocean if oceanic resonance amplifies the tidal response (Idini and Nimmo 2024). Future measurements by Dragonfly may shed new light on the tidal deformation and dissipation process. Charnay et al. (2022) showed that the DragMet pressure sensor may be accurate enough to detect gravitational atmospheric tides, thus providing new insights on the interior response, independent from Cassini measurements. Interpretation of the detailed morphology of the crater Selk and of related tectonic features could provide information on the flexural and relaxation processes, providing indirect constraints on the heat flux history and hence on internal heating. Again, further modeling and experimental efforts are needed to anticipate the interpretation of these future measurements.

## Footnotes

1 Analogous to the spherical harmonics $Y_{l}^{m}$, the order of the eigenfunction, $m$, refers to its integer wavenumber (see e.g. Varshalovich et al. 1988).
